# An evolutionary divergent thermodynamic brake in ZAP-70 fine-tunes the kinetic proofreading in T cells

**DOI:** 10.1016/j.jbc.2022.102376

**Published:** 2022-08-13

**Authors:** Kaustav Gangopadhyay, Arnab Roy, Athira C. Chandradasan, Swarnendu Roy, Olivia Debnath, Soumee SenGupta, Subhankar Chowdhury, Dipjyoti Das, Rahul Das

**Affiliations:** 1 Department of Biological Sciences, Indian Institute of Science Education and Research Kolkata, Mohanpur, India; 2 Centre for Advanced Functional Materials, Indian Institute of Science Education and Research Kolkata, Mohanpur, India

**Keywords:** kinase, cell signaling, kinetic model, T cell, proofreading, ZAP-70, BCR, B cell receptor, ITAM-Y2P, doubly-phosphorylated immunoreceptor tyrosine based activation motif, ITC, isothermal titration calorimetry, ODE, ordinary differential equation, PBP, phosphate-binding pocket, TCR, T cell receptor, tSH2, tandem Src homology 2

## Abstract

T cell signaling starts with assembling several tyrosine kinases and adapter proteins to the T cell receptor (TCR), following the antigen binding to the TCR. The stability of the TCR–antigen complex and the delay between the recruitment and activation of each kinase determines the T cell response. Integration of such delays constitutes a kinetic proofreading mechanism to regulate T cell response to the antigen binding. However, the mechanism of these delays is not fully understood. Combining biochemical experiments and kinetic modeling, here we report a thermodynamic brake in the regulatory module of the tyrosine kinase ZAP-70, which determines the ligand selectivity, and may delay the ZAP-70 activation upon antigen binding to TCR. The regulatory module of ZAP-70 comprises of a tandem SH2 domain that binds to its ligand, doubly-phosphorylated ITAM peptide (ITAM-Y2P), in two kinetic steps: a fast step and a slow step. We show the initial encounter complex formation between the ITAM-Y2P and tandem SH2 domain follows a fast-kinetic step, whereas the conformational transition to the *holo*-state follows a slow-kinetic step. We further observed a thermodynamic penalty imposed during the second phosphate-binding event reduces the rate of structural transition to the *holo*-state. Phylogenetic analysis revealed the evolution of the thermodynamic brake coincides with the divergence of the adaptive immune system to the cell-mediated and humoral responses. In addition, the paralogous kinase Syk expressed in B cells does not possess such a functional thermodynamic brake, which may explain the higher basal activation and lack of ligand selectivity in Syk.

The activation and quiescence in the cell-mediated immune response by T cell is regulated by a kinetic proofreading mechanism (1, 2, 3). According to this mechanism, a time delay separates the binding of an antigen to the T cell receptor (TCR) from the subsequent downstream signaling (4, 5, 6). TCR lacks intrinsic catalytic activity, and the downstream signaling starts with the recruitment of multiple kinases and adapter proteins to the complex (Fig. S1*A*) (7, 8, 9). Each recruitment step introduces a delay between the ligand binding and activation of the enzyme. The nonspecific interaction between self-antigen and TCR is short-lived and does not signal because the antigen–TCR complex dismantles before the activation of the downstream kinases. The interactions arising from foreign antigens are long-lived and get enough time to signal by activating the kinases. Paralogous kinases mediating the early events in B cell receptor (BCR) signaling of the humoral immune response employ conceptually similar mechanisms (10). Nevertheless, the mechanism of differential activation of early T cell signaling compared to B cell remains unclear (11, 12).

The Syk family of nonreceptor tyrosine kinases, ZAP-70 and Syk, are indispensable in the early stage of TCR and BCR signaling, respectively (10, 13). Both the kinases are activated by recruiting to the membrane following antigen binding (Fig. S1*A*). The dwell time of the kinases at the membrane determines their response (14). ZAP-70 and Syk, both shares a modular structure composed of an N-terminal regulatory module and a C-terminal kinase domain (Fig. 1*A*) (10, 13). The regulatory module is made up of tandem Src homology 2 (tSH2) domains connected by a helical linker called interdomain A (Fig. 1, *A* and *C*). In the inactive state (*apo*-state), the two SH2 domains adopt an ‘L’-like open conformation making them incompatible to ligand binding (Fig. 1*C*) (15, 16). The Syk kinases are activated by binding to the doubly-phosphorylated immunoreceptor tyrosine based activation motif (ITAM-Y2P) motifs at the TCR or BCR, respectively, through the tSH2 domain (Figs. 1*B* and S1*A*) (17, 18). The tSH2 domain adopts a closed conformation upon binding to ITAM-Y2P, releasing the autoinhibitory interactions leading to the activation of the kinase domain (Figs. 1*C* and S1*A*) (19, 20). The active conformation of ZAP-70 is stabilized by phosphorylating two key residues Y315 and Y319, by Lck, a Src family kinase, recruited to the antigen: TCR complex (21, 22, 23).

**Figure 1:**
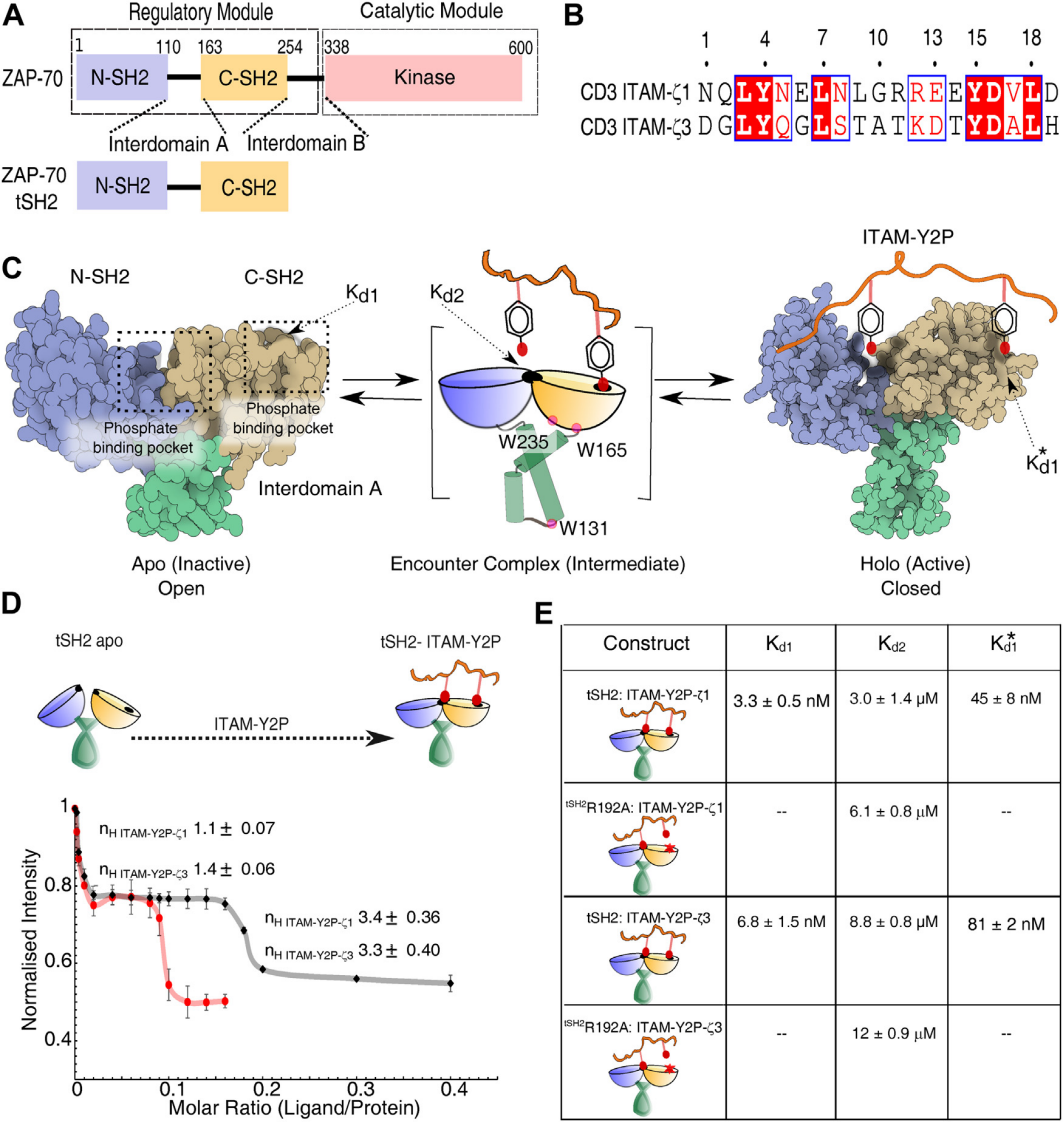
**Binding of tSH2 domain of ZAP-70 to the ITAM-Y2P peptides**. *A*, domain architecture of ZAP-70 full-length and the regulatory module. *B*, sequence alignment between ITAM-Y2P-ζ1 and ITAM-Y2P-ζ3 peptides. *C*, space-filled representation of tSH2-*apo* (PDB ID: 1M61) and tSH2-*holo* (PDB ID: 2OQ1) structure, the intermediate step is represented as cartoon. The N- and C-terminal SH2 domain, phosphate-binding pocket, and the respective binding constants are labeled. The tryptophan residues used for measuring intrinsic fluorescence are labeled. The residues are numbered according to the ZAP-70 *holo*-tSH2 domain structure (PDB ID: 2OQ1). *D*, plot of change in intrinsic tryptophan fluorescence as a function of indicated ITAM-Y2P: tSH2 domain ratio. The Hill coefficients were calculated using Hill Plot. The *solid red line* is for guiding eyes. The error bar represents the SD from three independent experiments. *E*, Table summarizes the respective binding constants for the indicated tSH2 domain and ITAM-Y2P. The $K_{d1}$ and $K_{d1}^{*}$ is reported from the intrinsic fluorescence titration and $K_{d2}$ is measured by ITC. The $K_{d1}^{*}$ values determined by ITC is tabulated in Table S3. Also see Fig. S1. tSH2, tandem Src homology 2; ITAM-Y2P, doubly-phosphorylated immunoreceptor tyrosine based activation motif; ITC, isothermal titration calorimetry.

Several lines of evidence suggest that the ZAP-70 and Syk behave differently in the cell. Notably, ZAP-70 does not display basal activation, whereas Syk-mediated basal signaling is essential for cell survival (24, 25, 26, 27, 28, 29). A ligand-independent closed conformation of the Syk tSH2 domain is proposed to facilitate high basal activation (30, 31). Despite the high sequence homology with Syk, it is not understood why the tSH2 domain of ZAP-70 could not adopt a stable closed conformation in the *apo*-state. It is also not clear why does ZAP-70 display a delayed Ca^2+^ response upon activation compared to Syk (12, 32). Moreover, the tSH2 domain of ZAP-70 binds in a biphasic manner with a high degree of selectivity to a conserved ITAM-Y2P sequence (33), compared to hyperbolic binding in Syk (34, 35, 36). The mechanism and functional significance of biphasic ligand binding for T cell signaling remains unclear.

We present a kinetic model from a comparative study of the tSH2 domain of ZAP-70 and Syk that explains the differential ligand binding. We observed that the tSH2 domain of ZAP-70 binds to ITAM-Y2P in two-step kinetics, fast and slow, compared to one-step binding in Syk. The slow binding to the ZAP-70 tSH2 domain arises from a thermodynamic penalty (brake) that determines the ligand selectivity and biases the conformational equilibrium of the *apo*-tSH2 domain toward the open conformation. Conversely, such thermodynamic break is nonfunctional in Syk tSH2-domain. Phylogenetic mapping shows that the emergence of the thermodynamic brake coincides with the evolution of the BCR-TCR-MHC like immune system at the divergence of jawless and jawed fish approximately 500 million years ago (37).

## Results and discussion

### The tSH2 domain of ZAP-70 is sensitive to the subtle changes in the ITAM peptide sequence

The ZAP-70 tSH2 domain binding to the doubly phosphorylated ITAM-ζ1 peptide (ITAM-Y2P-ζ1) produces a biphasic curve with three distinct dissociation constants, $K_{d1}$, $K_{d2}$, and $K_{d1}^{*}$ (Fig. 1, *B*–*D*) (33). First, the N-terminal phosphotyrosine residue from ITAM-Y2P binds uncooperatively to the C-SH2 phosphate-binding pocket (PBP) with a low nanomolar affinity ($K_{d1}$) to form an encounter complex (Fig. 1, *C*–*E*). The formation of the tSH2:ITAM-Y2P encounter complex allows the assembly of the N-SH2 PBP. Subsequently, C-terminal phosphotyrosine residue from ITAM-Y2P binds weakly to the newly formed PBP with micromolar affinity $\left(K_{d2}\right)$. In the steady-state, the two binding events are interlinked by a plateau (Fig. 1*D*). The second binding event remodels the C-SH2 PBP to an intermediate-binding pocket ($K_{d1}^{*}$) producing a *hill-coefficient* of 3.4 ± 0.36 (suggesting cooperative binding).

It was reported previously, the tSH2-domain of ZAP-70 displays hierarchical preference in binding to different ITAM sequences (17, 35, 38, 39, 40). We begin by asking which part of the biphasic binding isotherm, in the steady-state, is sensitive to the ITAM peptide sequence (Fig. 1*B*). We comparatively studied the binding of ITAM-Y2P-ζ3 to the tSH2 domain by intrinsic tryptophan fluorescence spectroscopy and isothermal titration calorimetry (ITC) (Figs. 1, *B*, *D*–*E* and S1, *B*–*F*). We probed $K_{d1}$ and $K_{d1}^{*}$ by fluorescence spectroscopy and $K_{d2}$ and $K_{d1}^{*}$ by ITC. To probe the $K_{d2}$, we used ^tSH2^R190A mutant that impairs phosphotyrosine binding to C-SH2 PBP (Figs. 1*E* and S1*F*).

We overserved that ITAM-Y2P-ζ3 binds weakly to the ZAP-70 tSH2 domain, compared to ITAM-Y2P-ζ1, consistent with the previous reports (17, 41) (Fig. 1*E*). Our data revealed that the C-SH2 domain does not distinguish between the two ITAM-Y2P while forming the encounter complex. Both the peptides, ITAM-Y2P-ζ1 and ITAM-Y2P-ζ3, bind to the C-SH2 domain with low nanomolar affinity ($K_{d1}$) of 3.3 ± 0.5 nM and 6.8 ± 1.5 nM, respectively. However, we noted a significant increase in the plateau width for the ITAM-Y2P-ζ3 and tSH2 interaction (Fig. 1*D*). The ITAM-Y2P-ζ3 binding perturbed the $K_{d2}$ and $K_{d1}^{*}$ contributing to the overall increase in the dissociation constant (Fig. 1*E*). We ask why the plateau-width in the steady-state binding (Fig. 1*D*) is sensitive to the subtle changes in ligand type?

### A multistep ligand-receptor model explains the binding kinetics of ITAM-Y2P to ZAP-70

**Figure 2:**
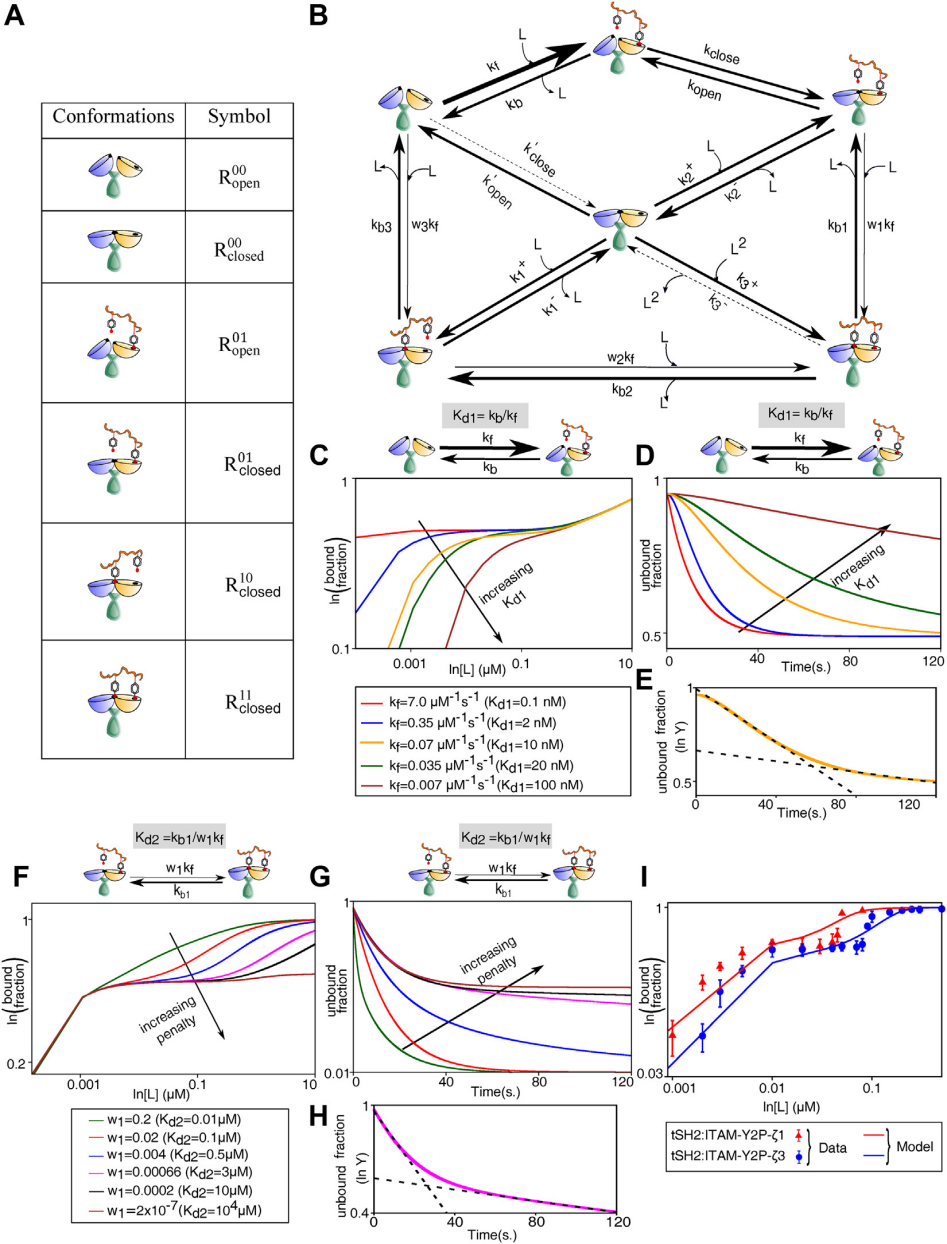
**A kinetic-mathematical model explaining the biphasic ligand binding.***A*, receptor conformations used in the model and their corresponding symbols. *B*, schematic diagram of the model showing different reaction pathways. Arrow-widths represent distinct weightages of kinetic rates (*bold arrows*: higher rates, *thin arrows*: lower rates, and *dotted arrows*: negligible rates). *C*, theoretical curves of the bound fractions, defined as the proportion of partially bound closed states and the holo-state (${\text{R}}_{\text{closed}}^{01}$, ${\text{R}}_{\text{closed}}^{10}$, and ${\text{R}}_{\text{closed}}^{11}$ ; see Equation S2), is plotted against the ligand concentration. In the steady-state, the effect of $K_{d1}$ variation is shown, while other parameters are kept constant as in Table S4. *D*, in the presteady state condition, the representative kinetic profiles showing the effect of $K_{d1}$ variation on the unbound fraction. *E*, two-step decay in the kinetic profile is shown with two distinct exponential fits (*dashed line*) in a semi-log plot. *F* and *G*, effects of $K_{d2}$ variation on the bound and unbound fraction under steady-state and presteady state, respectively (other parameters are fixed as in Table S4). *H*, similar to panel (*E*), two exponential fits (*dashed line*) represent distinctive two-step kinetics. *I*, comparison of theoretical prediction with the experimental data for binding of indicated ITAM-Y2P to the ZAP-70 tSH2 domains. Fitted parameters are in Table S5. All kinetic plots in the model are shown under the saturating ligand concentration (*i.e.*, $\text{L}\gg {\text{R}}_{\text{open}}^{00}$; exact concentration values are mentioned in the Experimental procedures). Also see Fig. S2. tSH2, tandem Src homology 2; ITAM-Y2P, doubly-phosphorylated immunoreceptor tyrosine based activation motif.

We developed a multistep mathematical-kinetic model to explain the biphasic bindings of ITAM-Y2P and tSH2 domains. This model comprised of different tSH2 domain conformations, open and closed, connected by a complex network (Fig. 2, *A* and *B*). The tSH2-*apo* state ($R_{open}^{00}$) ultimately reaches the tSH2-*holo* state ($R_{closed}^{11}$) through different pathways associated with distinct rates. The receptor in the *apo*-state first converts to an encounter complex ($R_{open}^{01}$) and then adopts a closed conformation ($R_{closed}^{01}$). We also considered two other intermediates ($R_{closed}^{00}$ and $R_{closed}^{10}$) through which the final *holo*-state may form (see Experimental procedures). Based on our experimental dissociation constants, we assumed that the formation of the encounter complex is the fastest ($K_{d1}=\frac{k_{b}}{k_{f}}=3-10\;nM$; Fig. 1*E*). To explain the biphasic binding (Fig. 1*D*), we further assumed that the transitions to the *holo*-state from the partially bound states (from $R_{closed}^{10}$ or $R_{closed}^{01}$ to $R_{closed}^{11}$) exhibit negative cooperativity. These steps represent kinetic penalties (42) and occur with much slower forward rates ($w_{1}k_{f}$ and $w_{2}k_{f}$, with $0<w_{i}<1$ , i=1 or 2) with dissociation constants $K_{d2}(=\frac{k_{b1}}{w_{1}k_{f}})$ and $K_{d1}^{*}$ (= $\frac{k_{b2}}{w_{2}k_{f}}$), respectively. It may be noted that our model does not have any feedback regulation (43, 44) and is based on allosteric interaction between the two SH2 domains (33). We anticipate the variation in $K_{d1}$, $K_{d2}$, or $K_{d1}^{*}$ may determine the steady-state response.

The model was analyzed numerically to calculate the ligand-bound fraction (Equations S1 and S2) and to predict the steady-state response and the kinetic behavior. In the steady-state, the $K_{d1}$ determines the receptor sensitivity (initial rising slopes of the bound fractions) in low ligand concentration regime (nM level) and also modulates the plateau width (Fig. 2*C*). The kinetic behavior mostly showed a single exponential decay except in a narrow intermediate range of $K_{d1}$ (around 8 nM – 20 nM), where a two-step decay was observed (Figs. 2, *D* and *E* and S2, *A* and *B*).

Next, we assessed the effect of $K_{d2}$ (Fig. 1*C*) variation on the steady-state behavior by changing the penalty factor, $w_{1}$ (associated with the transition from $R_{closed}^{01}$ to $R_{closed}^{11}$, Fig. 2*B*). The $K_{d2}$ variation modulated the ligand selectivity by altering the plateau width in the steady-state (Fig. 2*F*). With a higher penalty (equivalently lower $w_{1}$ or higher $K_{d2}$), the plateau width became broader and displayed two-step kinetics with a sharp initial decay and a slow subsequent decrease of the unbound fraction. (Fig. 2, *G* and *H*).

We asked, does a biphasic behavior in the steady-state arise due to slow transition of the partially-bound closed states ($R_{closed}^{01}$ or $R_{closed}^{10}$) to an open configuration. In our model, we introduced slow transition from $R_{closed}^{01}$ or $R_{closed}^{10}$ states to open configurations $(R_{open}^{01}$ or $R_{open}^{00},\text{respectively})$ by varying the respective rates, at the same time without putting penalties on other steps ($K_{d2}$ and $K_{d1}^{*}$). None of the changes made produced any biphasic response in the steady-state (Fig. S2, *E*–*H*). In contrast, when we introduce slow transition from $R_{closed}^{01}$ or $R_{closed}^{10}$ to the final *holo*-state ($R_{closed}^{11}$), the steady-state responses become biphasic (Fig. S2, *I*–*L*). Thus, we conclude that the slow transitions (due to the penalty) from the $R_{closed}^{01}$ and $R_{closed}^{10}$ to the $R_{closed}^{11}$ determines the plateau behavior and not the slow relaxation of the close states to the open state.

Since both the slow transition from the $R_{closed}^{01}$ or $R_{closed}^{10}$ states to the final *holo*-state ($R_{closed}^{11}$) could, in principle, lead to a biphasic response, we next asked which transition is more sensitive. We found that the variation of $K_{d1}^{*}$ marginally alters the plateau width in the steady-state (Fig. S2, *C* and *D*) (when we introduced penalties in the transitions from $R_{closed}^{10}$ and $R_{closed}^{01}$ to $R_{closed}^{11}$). Therefore, the slowest binding step to the N-SH2 PBP ($K_{d2}$) mainly controls the plateau width. This conclusion correlates with the observed $K_{d2}$, which is orders of magnitude lower than the $K_{d1}$ and $K_{d1}^{*}$ (Fig. 1*E*), suggesting the corresponding step may impart a significant penalty in the dynamic binding of the ligand. However, measurement of free energy change is necessary to determine if a thermodynamic cost manifests as a kinetic penalty, as elucidated in the model.

Finally, for a particular set of parameter choices, the model prediction reasonably agreed with the experimental data of ITAM-Y2P-ζ1 and ITAM-Y2P-ζ3 bindings to the tSH2 domain (Fig. 2*I*). In the model, reported values of $K_{d1}$, $K_{d2}$, and $K_{d1}^{*}$ (Fig. 1*E*) were used for the quantitative matching, but other parameters were unknown and chosen arbitrarily to fit the data (Table S5).

### The tSH2 domain of ZAP-70 binds ITAM-Y2P in two kinetic steps

**Figure 3:**
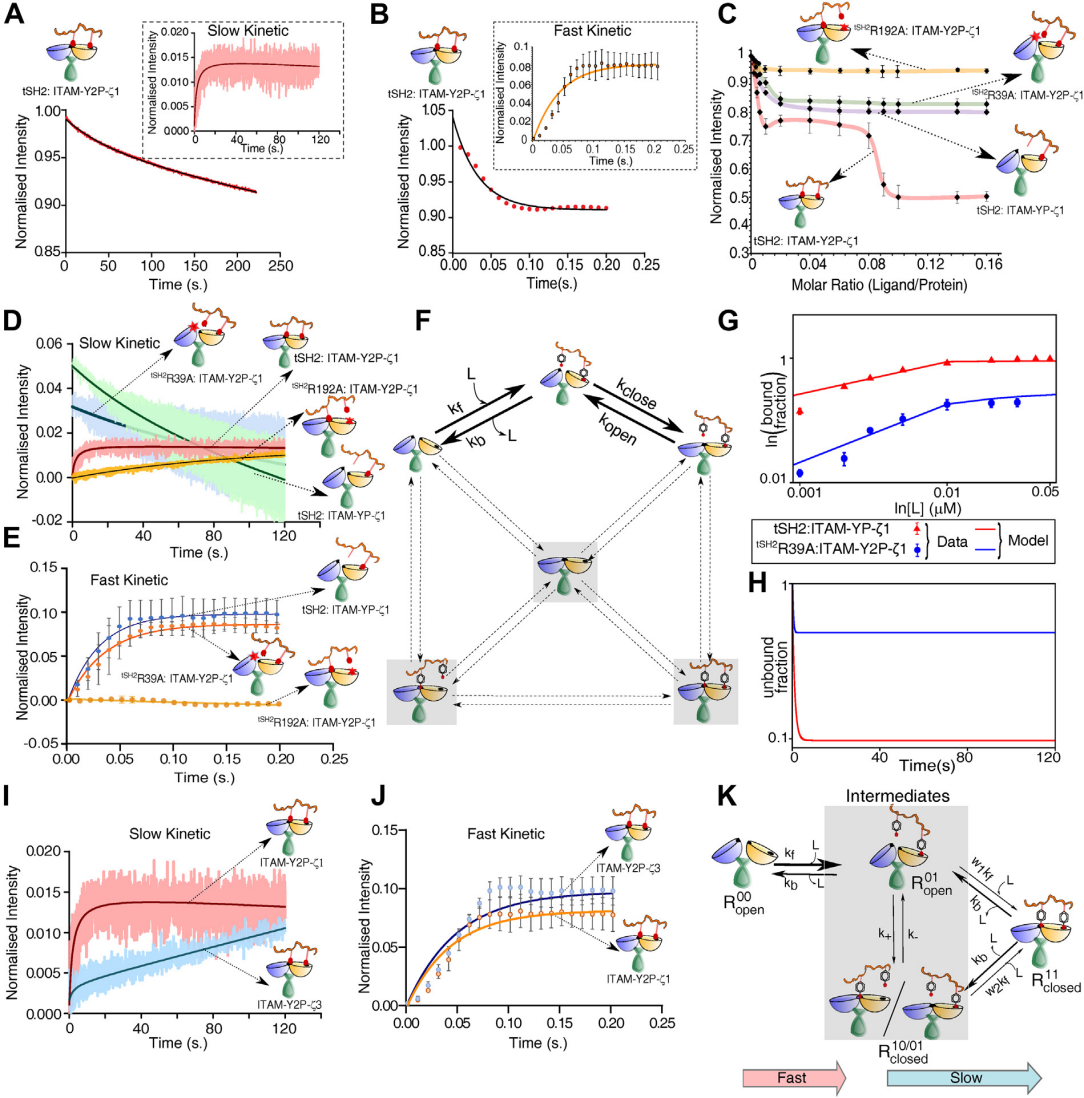
**Binding kinetic of tSH2 domain of ZAP-70 and ITAM-Y2P**. *A* and *B*, are the plots of time-dependent binding kinetics of tSH2 domain (conc. of 100 nM) and ITAM-Y2P-ζ1 (conc. of 30 μM) in the slow and fast time scales, respectively. Inset represents the single exponential fitting of the blank subtracted data. The error bar represents the SD from three independent experiments. *C*, the steady-state binding of the indicated tSH2 construct to ITAM-Y2P-ζ1 and ITAM-YP-ζ1. The *solid red line* is for guiding eyes. *D* and *E*, are the plots of slow and fast binding kinetics of indicated tSH2 construct to ITAM-Y2P-ζ1 and ITAM-YP-ζ1, respectively. The residues are numbered according to the ZAP-70 *holo*-tSH2 domain structure (PDB ID: 2OQ1). *F*, a modified version of the full model (Fig. 2*B*) to explain the binding kinetics of ^tSH2^R39A to ligand. Forbidden intermediate conformations are highlighted by *shadow boxes* and *dotted arrows*. *G* and *H*, the bound and unbound fractions of tSH2:ITAM-YP and ^tSH2^R39A:ITAM-Y2P under the steady-state and presteady state conditions, respectively. In panel (*G*), experimental steady-state data are also shown for comparison. *I* and *J*, binding kinetics of ITAM-Y2P-ζ1 and ITAM-Y2P-ζ3 to the ZAP-70 tSH2 domain. The error bar represents the SD from three independent experiments. *K*, a reduced kinetic model of ligand binding derived from the full model (Fig. 2*B*). The reduced model neglects the formation of ligand-independent closed state (${\text{R}}_{\text{closed}}^{00}$) and assumes that the formation of encounter complex (${\text{R}}_{\text{open}}^{01}$) is critical for subsequent binding steps. Also see Fig. S3. tSH2, tandem Src homology 2; ITAM-Y2P, doubly-phosphorylated immunoreceptor tyrosine based activation motif.

Since our model predicted two-step binding kinetics (Fig. 2, *E* and *H*), we next probed the binding kinetics of ITAM-Y2P-ζ1 or ITAM-Y2P-ζ3 to the tSH2 domain by stopped-flow fluorescence spectroscopy. We started with mixing excess ITAM-Y2P-ζ1 to the tSH2 domains at 10 °C and measured the change in tryptophan fluorescence intensity for 200 s (Fig. 3*A*). We observed that the fluorescence intensity decay in two steps, fast (<200 ms) and slow (>20 s) (Fig. 3*A*). Hence, we recorded all the kinetic experiments at two-time scales. All kinetic data were first normalized against the highest intensity and then subtracted by the blank (protein only sample) (Fig. 3, *A* and *B*) and fitted to a one-site association kinetics (Table 1).

**Table 1:** Observed rate for the ZAP-70 tSH2 domain and ITAM-Y2P binding

Construct	$k_{obs}^{fast}$ (s^-1^)	$k_{obs}^{slow}$ (s^-1^)
tSH2:ITAM-Y2P-ζ1	23.87 ± 1.01	0.262 ± 0.0977
tSH2:ITAM-Y2P-ζ3	21.44 ± 1.83	0.012 ± 0.0048
^tSH2^ R39A:ITAM-Y2P-ζ1	24.82 ± 2.29	---
tSH2:ITAM-YP-ζ1	28.60 ± 5.84	---
tSH2: ^ITAM-Y2P-ζ1^ E13A	24.4 ± 2.04	0.022 ± 0.0045
^tSH2^ F117A: ITAM-Y2P-ζ1	21.83 ± 1.49	0.0517 ± 0.0062
^tSH2^ R43P: ITAM-Y2P-ζ1	27.96 ± 3.45	2.25 ± 0.032
^tSH2^ R192A: ITAM-Y2P-ζ1	---	0.006 ± 0.002

We observed that the ITAM-Y2P-ζ1 binds to the tSH2 domain with two observed rates of $k_{obs}^{fast}=23.87\pm 1.01$ s^−1^ and $k_{obs}^{slow}=0.262\pm 0.098$ s^−1^ (Fig. 3, *A*, *B* and Table 1). Our mathematical model suggests that the fast-binding kinetic may arise (Fig. 2, *E* and *H*) during the formation of the encounter complex ($K_{d1}$). To test that, we turn to three samples, two mutants ^tSH2^R39A and ^tSH2^R192A that would prevent phosphotyrosine binding to N-SH2 and C-SH2 PBP, respectively. Third, a single phosphotyrosine ITAM-ζ1 peptide (ITAM-YP-ζ1) that will show only one binding event (Fig. 3*C*). The steady-state fluorescence titration of ^tSH2^R39A to ITAM-Y2P-ζ1 and tSH2 domain to ITAM-YP-ζ1 showed that the first binding step is preserved with ($K_{d1}$) of 8 ± 1.05 nM and 3.7 ± 0.1 nM, respectively, and no subsequent binding was observed (Figs. 3*C*, S3, *A* and *B*). In the kinetics experiment, both the samples showed a $k_{obs}^{fast}$ of 24.82 ± 2.29 s^−1^ and 28.60 ± 5.84 s^−1^, respectively, with no detectable slow binding (Fig. 3, *D* and *E*). Under substoichiometric ligand concentration, ^tSH2^R192A did not bind to the ITAM-Y2P-ζ1(33) and showed a linear ligand binding at a higher ITAM-Y2P-ζ1 (μM) concentration (Fig. S3*G*). The ^tSH2^R192A binds with a ten-fold slower $k_{obs}^{slow}$ (0.006 ± 0.002 s^−1^) rate in comparison to the WT tSH2 domain (Table 1), with no detectable fast binding (Fig. 3, *D* and *E*). Together, our data indicates that the N-SH2 (R39A) and C-SH2 (R192A) mutants display only μM and nM affinity, respectively, suggesting that the formation of the encounter complex is critical for the subsequent ligand binding.

To check if our proposed model (Fig. 2*B*) could explain the above data (Fig. 3, *C*–*E*), we introduced a modification in the model (Fig. 3*F*). The N-SH2 binding is almost absent in both cases, and the partially bound state does not transform to the final *holo*-state. Hence, we ignored all pathways except the first encounter complex and the subsequent conformational change (Fig. 3*F*). This assumption was sufficient for a quantitative matching between the model prediction and the experimental data in the steady-state (Fig. 3*G*). The corresponding kinetic behavior also showed a single exponential decay as observed in our experiment (Fig. 3*H*).

Our model suggests that the tSH2 domain forms the encounter complex $\left(K_{d1}\right)$ with a fast-kinetic step, whereas the phosphate binding to the N-SH2 PBP ($K_{d2}$) is the rate-limiting step and may determine the plateau width (Fig. 2, *F*–*H*). To validate, we measured the binding kinetics of the tSH2 domains and ITAM-Y2P-ζ3 (Fig. 1*D*). Indeed, the ITAM-Y2P-ζ3 binds slower ($k_{obs}^{slow}$ = 0.012 ± 0.005 s^−1^) to the N-SH2 PBP than ITAM-Y2P-ζ1, with no significant change in the fast-kinetic step (Figs. 2*I*, 3,*I* and *J* and Table 1).

Our experiments suggest that (i) the $K_{d2}$ is the rate-limiting step, (ii) the formation of the encounter complex ($R_{open}^{01}$) is essential for the transition to the *holo*-state, and (iii) a direct transition to the *holo*-state through the species $R_{closed}^{10}$ and $R_{closed}^{00}$ is unlikely. Therefore, a reduced version of the full model (Fig. 2*A*) is sufficient to capture essential features of the two-step kinetics (see Experimental procedures). This reduced model has three basic steps: (i) a fast encounter to C-SH2 pocket (ii) followed by slower conformational changes of intermediates (open ↔ closed) and (iii) subsequent transition of intermediates to the final *holo*-state by imposing a kinetic penalty (Fig. 3*K*). The minimal model was solved mathematically, and it produced qualitatively the same results as the full model (see Equations S4 and S5 and Fig. S3, *C*–*F*). However, incorporating the finer details in the full model was necessary for quantitative matching with the data. We now ask, what determines the nature of the slow-kinetic step?

### The nature of the slow-kinetic step is determined by the structural coupling between the two SH2 domains

**Figure 4:**
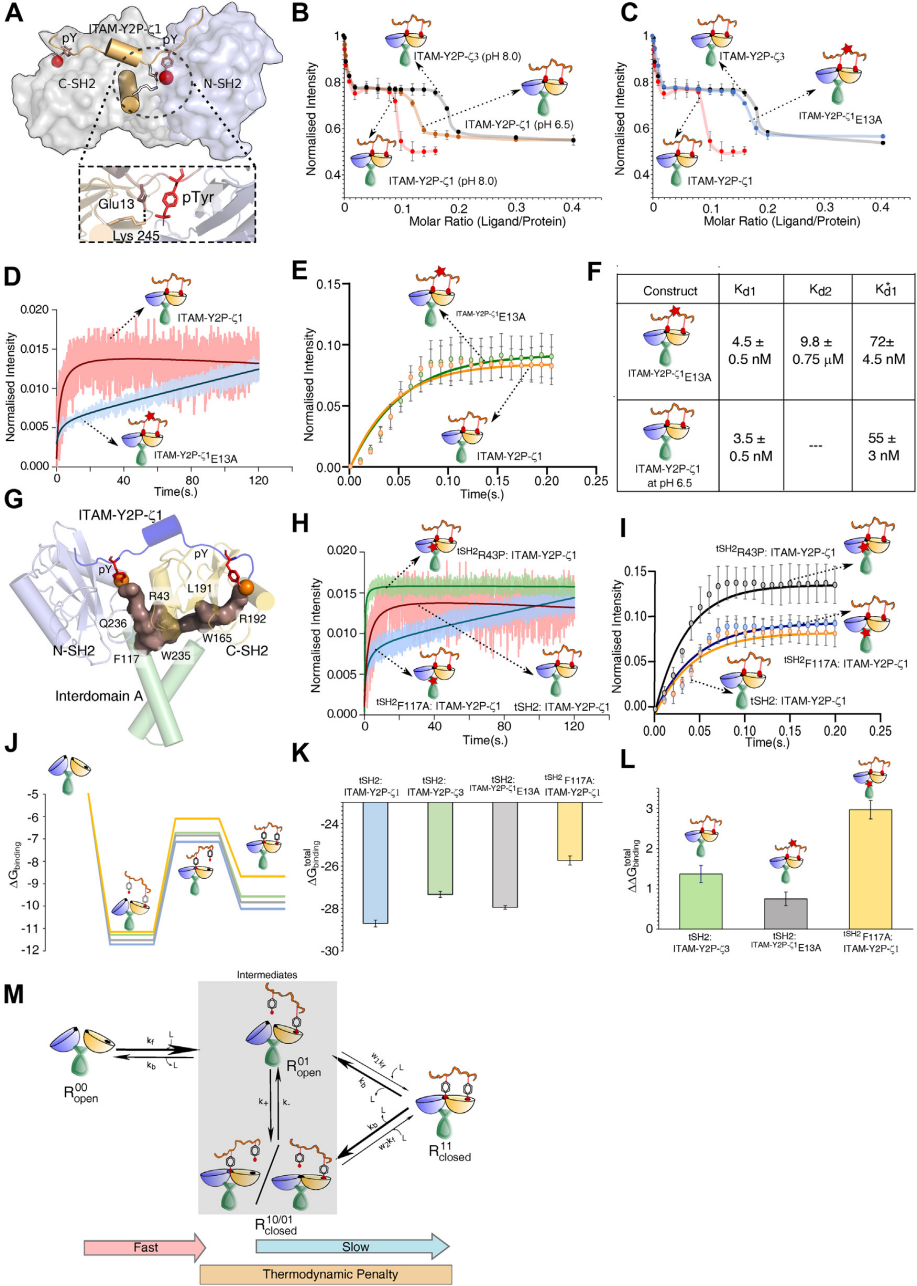
**Structural evaluation of the tSH2 domain in determining the penalty step**. *A*, space-filled model of tSH2-*holo* structure of ZAP-70 (PDB ID: 2OQ1) highlighting the salt-bridge between the tSH2 domain and ITAM-Y2P-ζ1. *B* and *C*, the steady-state binding of the tSH2 domain to ITAM-Y2P-ζ1 at indicated pH and ITAM-Y2P-ζ1 mutant, respectively. The *solid red line* is for guiding eyes. The error bar represents the SD from three independent experiments. *D* and *E*, the plots of slow and fast binding kinetics of tSH2 domain to indicated ITAM-Y2P-ζ1 peptides, respectively. The error bar represents the SD from three independent experiments. *F*, table summarizing the dissociation constants for the indicated tSH2 domain and ITAM-Y2P samples. The $K_{d1}$ and $K_{d1}^{*}$ is reported from the intrinsic fluorescence titration and $K_{d2}$ is measured by ITC. *G*, structure representing the allosteric network coupling the two SH2 domains in ZAP-70 (PDB ID: 2OQ1). *H* and *I* slow and fast binding kinetics of indicated tSH2 domain mutants, respectively. The error bar represents the SD from three independent experiments. *J*, represents the change in Gibb’s free energy upon ligand binding ($\Delta G_{binding}$) in tSH2:ITAM-Y2P-ζ1 (*blue*), tSH2:ITAM-Y2P-ζ3 (*green*), tSH2:^ITAM-Y2P-ζ1^E13A (*gray*), and ^tSH2^F117A:ITAM-Y2P-ζ1 (*yellow*). *K* and *L* are the $\Delta G_{binding}^{total}$ and $\Delta \Delta G_{binding}^{total}$, for the indicated tSH2 domain: ITAM-Y2P interactions. The error bar represents standard error. *M*, schematic model showing the binding kinetics and the thermodynamic penalty in the tSH2 and ITAM-Y2P interaction. Also see Fig. S4. tSH2, tandem Src homology 2; ITAM-Y2P, doubly-phosphorylated immunoreceptor tyrosine based activation motif; ITC, isothermal titration calorimetry.

The ‘open to closed’ structural transitions of ZAP-70 tSH2 domain upon ligand binding requires cooperative interaction between the ITAM peptide, and the allosteric network resides in the tSH2 domain (Fig. 1*C*) (17, 33, 45). Analyzing the crystal structure of ZAP-70 tSH2 domain in complex with ITAM-Y2P-ζ1 (19), we identified a salt-bridge between the ^ITAM-ζ1^E13 and ^tSH2^K245 residue that may be critical for the structural coupling between the N- and C-SH2 domain during ligand binding (Fig. 4*A*). The corresponding residue in ITAM-ζ3 is an aspartic acid, which may increase the distance between the ion-pair in the salt-bridge. This may reduce the transition rate to the final *holo*-state $\left(k_{obs}^{slow}\right)$ and increase the plateau width in the steady-state (Figs. 1, *B*, *D*, and 3*I*). To test the role of the salt-bridge in determining the plateau width, we modulated the strength of the salt-bridge by changing the pH or by ^ITAM-ζ1^E13A mutation (Figs. 4, *A*–*E*, S4*D* and S1*B*). We observed that lowering the pH to 6.5 changes the surface potential of the tSH2 domain (Fig. S4*D*) and marginally increases the plateau width, suggesting that the ^ITAM-ζ1^E13 and ^tSH2^K245 salt-bridge may be essential in the structural coupling (Fig. 4*B*). To further investigate the role of the salt bridge, we measured the steady-state and kinetics of the tSH2 domain binding to the ^ITAM-ζ1^E13A. We observed that the ^ITAM-ζ1^E13A increases the plateau width in the steady-state binding with a significant increase in the $K_{d2}$ and $K_{d1}^{*}$ compared to the ITAM-Y2P-ζ1 (Figs. 1*E* and 4, *C*–*F*). The binding kinetics shows that the mutation in the ITAM-Y2P-ζ1 does not perturb the rate of encounter complex formation ($k_{obs}^{fast}$) but slows down the transitioning to the closed-conformation ($k_{obs}^{slow}$ = 0.022 ± 0.005 s^−1^), similar to ITAM-Y2P-ζ3 binding (Figs. 3,*I*, *J*, 4, *D* and *E*, and Table 1). Our data suggest that the coupling between the two SH2 domains may determine the rate of the slow kinetic step ($k_{obs}^{slow}$).

A network of residues comprised of aromatic–aromatic stacking interaction allosterically couple the two SH2 domains of ZAP-70 during the transition to the final *holo*-conformation (Fig. 4*G*) (33). Mutating the residues in the allosteric network uncouples the formation of the encounter complex to the phosphotyrosine binding to the N-SH2 PBP ($K_{d2}$). To determine if the mutation in the allosteric network will reduce the rate of conformational transition to the closed state ($k_{obs}^{slow}$), we studied the effect of ^tSH2^R43P and ^tSH2^F117A mutants on the ITAM-Y2P-ζ1 binding kinetics (Fig. 4,*G*–*I* and Table 1). We observed that the allosteric mutant did not perturb the rate of encounter complex formation ($k_{obs}^{fast}$) compared to the WT tSH2 domain. As anticipated, the allosteric mutants, ^tSH2^R43P and ^tSH2^F117A, either inhibit or reduce the $k_{obs}^{slow}$, respectively (Fig. 4,*H* and *I* and Table 1). Analysis of the change in Gibb’s free-energy due to ligand binding ($\Delta G_{binding}$) suggests that the final transition to the close-conformation may impart a thermodynamic penalty (Fig. 4, *J*–*L*). Therefore, weakening the allosteric coupling led to a higher penalty at the transitions to the closed-state (Fig. 3*K*). We conclude that the residues in the allosteric network collectively constitute a thermodynamic brake which may impart a delay between ZAP-70 binding and activation at the membrane (Fig. 4*M*). It is widely believed that TCR shares a common ancestry and design principle with the BCR. Is the thermodynamic brake present in Syk?

### Thermodynamic penalty coincides with the evolutionary-divergence of humoral and cell-mediated immune response

**Figure 5:**
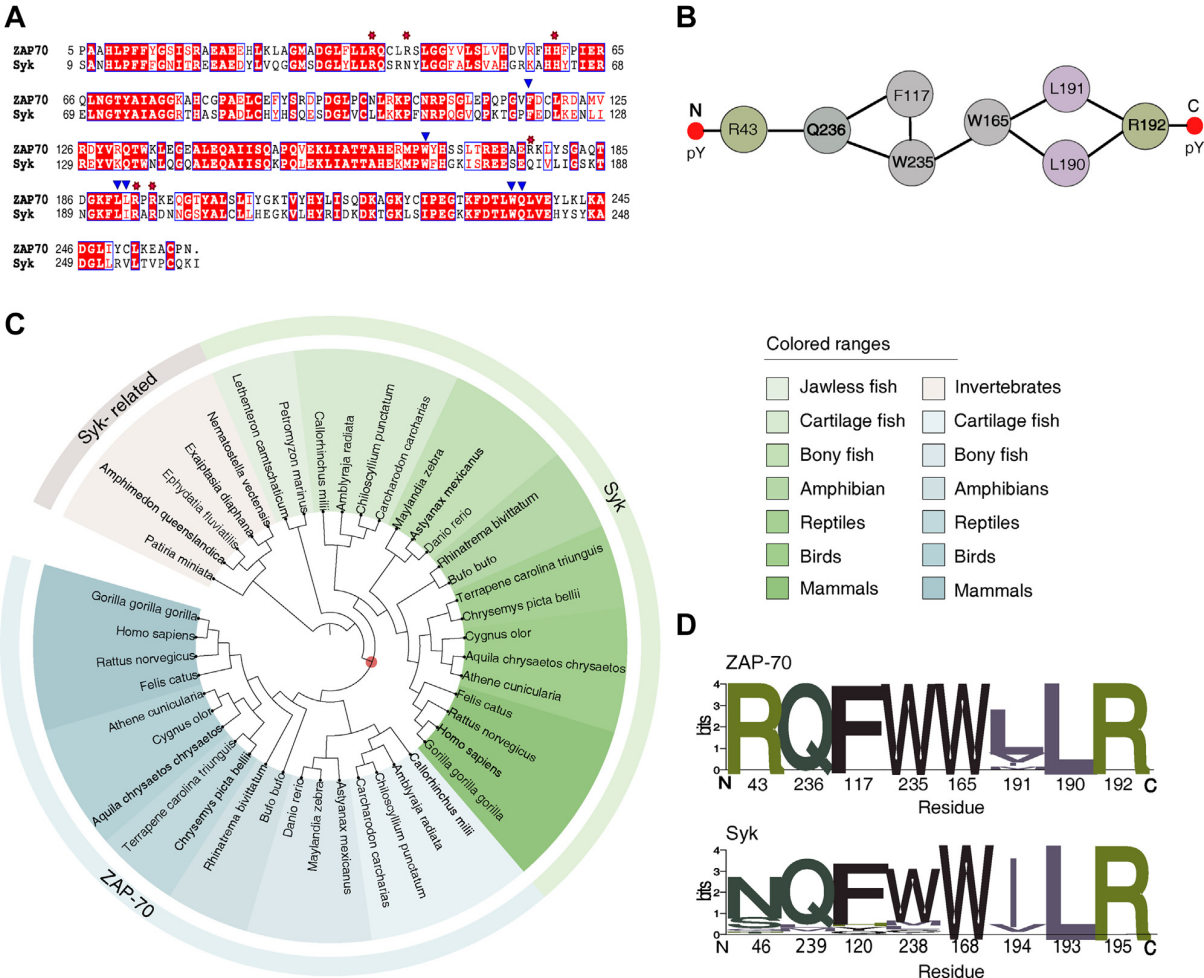
**Phylogeneticanalysisof allosteric network in the Syk family kinases***A*, sequence alignment of tSH2 domain of Syk and ZAP-70. The residues at the PBP are marked with *red-star*, and the allosteric network residues are marked with *blue-arrow*. The residues are numbered according to the ZAP-70 and Syk tSH2 domain structure, PDB ID: 2OQ1 and 1A81, respectively. *B*, schematic representation of undirected allosteric network in the tSH2 domain of ZAP-70. *C*, phylogenetic tree depicting the evolution of Syk family kinases and Syk-related kinases. The emergence of ZAP-70 is marked as a *red dot*. *D*, sequence logo representing the conservation of allosteric network residue in ZAP-70 (*top*) and in Syk (including the Syk-related kinases) (*bottom*). Also see Tables S1, S2, Figs. S5 and S6. tSH2, tandem Src homology 2; PBP, phosphate-binding pocket.

Syk is less selective than ZAP-70 and activated by a wide range of ITAM sequences in cells of innate and adaptive immune systems (10, 17, 20, 36, 46, 47, 48). Sequence analysis shows that most PBP and allosteric network residues in the regulatory module are conserved between Syk and ZAP-70 (Fig. 5, *A* and *B*). The exception is ^ZAP-70^R43; the corresponding residue in Syk is glutamine (Fig. 5, *A* and *B*). Phylogenetic mapping using the ZAP-70 as reference revealed that the ZAP-70 is conserved in all vertebrates and may appear first in the jawed fish (cartilage fish) (Fig. 5*C*) (30, 49). Syk and Syk-related proteins are present in vertebrates and some invertebrates, including sponges and hydra (Figs. 5*C* and S6*A*). This indicates that ZAP-70 may appear along with the evolution of the BCR-TCR-MHC like adaptive immune system at the divergence of jawless and jawed fish (37, 50). Intriguingly, the allosteric network residues comprising the thermodynamic brake in ZAP-70 are conserved, except residue L191 (Fig. 5, *B*–*D*). In amphibians and jawed fish, the Leu is often replaced by similar residues like Ile, Val, and Met. In Syk, two key residues in the allosteric network, N46 and W238 (corresponds to ZAP-70 R43 and W235, respectively), are not conserved (Figs. 5, *B*–*D* and S6*B*), suggesting that the thermodynamic brake may be nonfunctional.

**Figure 6:**
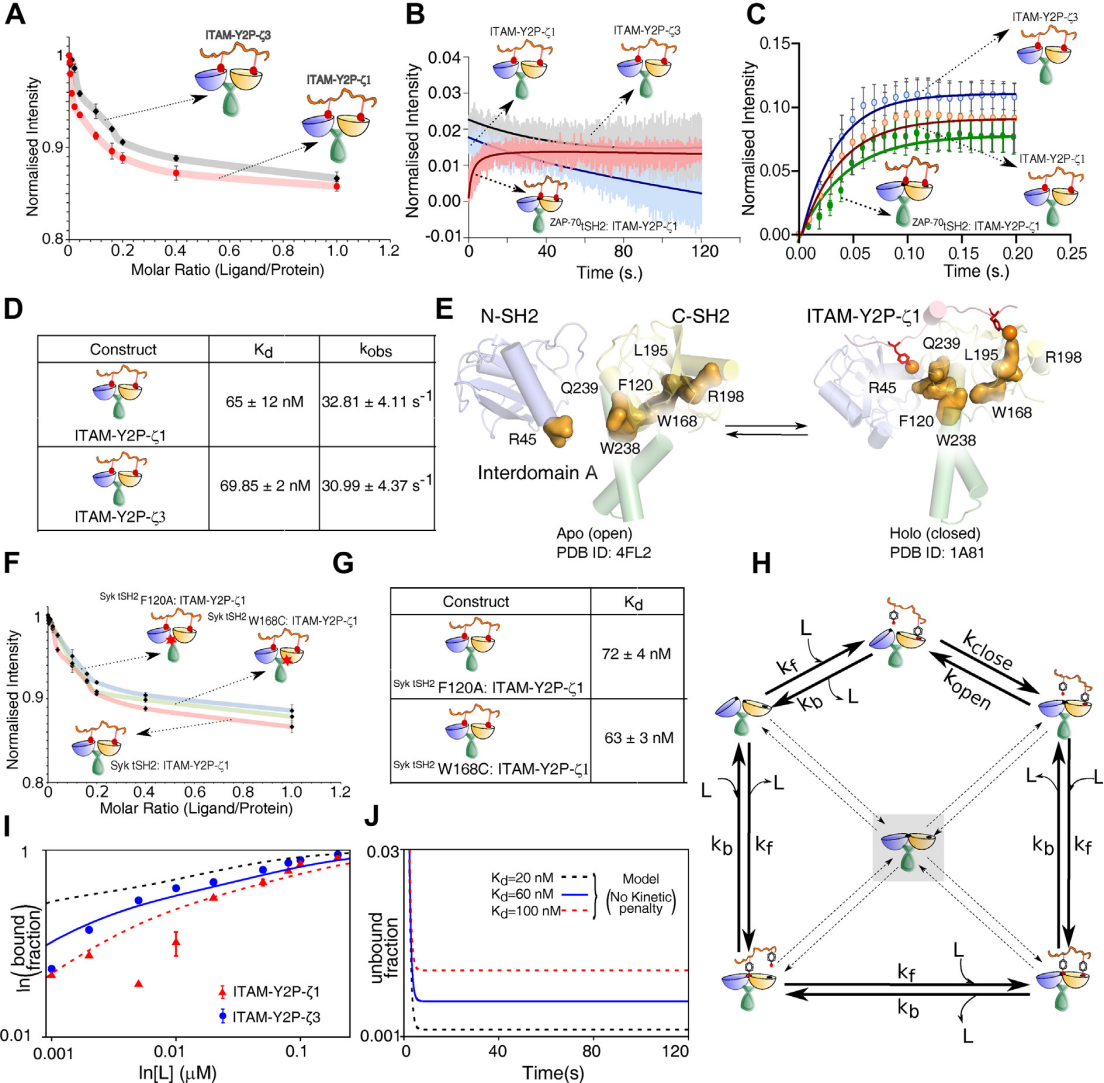
**Steady-state and kinetic binding of Syk tSH2 domain to ITAM-Y2P**: *A*, steady-state binding of ITAM-Y2P-ζ1 and ITAM-Y2P-ζ3 to the Syk tSH2 domain measured by intrinsic tryptophan fluorescence. The *solid red line* is for guiding eyes. The error bar represents the SD from three independent experiments. *B* and *C*, slow and fast binding kinetics of indicated tSH2 domains to ITAM-Y2P peptides. *D*, table summarizing the dissociation constant $\left(K_{d}\right)$ and observed rate constant $\left(k_{obs}\right)$ for the Syk tSH2 domain. *E*, structure highlighting the allosteric network in the Syk tSH2 domain in *apo*- (PDB ID: 4FL2) and *holo-*state (PDB ID: 1A81), respectively. The residues are numbered according to the Syk *holo*-tSH2 domain structure (PDB ID: 1A81). *F* steady-state binding of the indicated tSH2 construct to ITAM-Y2P-ζ1. *G*, tabulation of dissociation constant $\left(K_{d}\right)$ for the tSH2 allosteric mutant. The error bar represents the SD from three independent experiments. *H*, a modified version of the full model (Fig. 2*B*) explaining Syk ITAM–Y2P interaction. The modified model assumes no kinetic penalty at any step and neglects the formation of ligand-independent closed state (${\text{R}}_{\text{closed}}^{00}$ ; highlighted by *shadow boxes* and *dotted arrows*). *I*, comparison of the predicted bound-fraction with the experimental data (filled *circles* and *triangles*) in the steady-state. *J*, presteady state kinetic profiles of the unbound fraction showing single-step decay. Also see Fig. S7. tSH2, tandem Src homology 2; ITAM-Y2P, doubly-phosphorylated immunoreceptor tyrosine based activation motif.

To evaluate the role of the thermodynamic-brake in the Syk tSH2 domain, we characterized the steady-state binding of the tSH2 domain to ITAM-Y2P-ζ1 and ITAM-Y2P-ζ3, respectively. As reported previously, we observed that the Syk tSH2 domain could not distinguish between the ITAM sequences and bind with a similar *K*_*d*_ (∼65 nM) (Fig. 6, *A* and *B*) (36). Unlike ZAP-70, the tSH2 domain of Syk binds ITAM-Y2P in a single fast kinetic step ($k_{obs}^{fast}$ = 32.81 ± 0.005 s^−1^), without any subsequent slow binding (Fig. 6, *B*–*D*). To evaluate if the absence of this slow binding correlates with the integrity of the allosteric network (33), we inspected the open and closed structures of the Syk tSH2 domain (Figs. 4*G* and 6*E*). Analysis of the crystal structures showed that the ZAP-70 tSH2 domain could adopt only two conformations, *open (apo)* and closed (holo) (Fig. 1*C*) (19, 31). In comparison, the Syk tSH2 domain adopts three conformations: two structures in the *apo*-state, open and closed, and one ITAM-Y2P–bound closed conformation (16, 20, 30). In the apo-state of both Syk and ZAP-70, the SH2 domains are separated, preventing the aromatic amino acid residues to form the stacking interaction, which is central in coupling the N- and C-SH2 domains (Figs. 4*G* and 6*E*). Surprisingly, the aromatic–aromatic stacking interaction does not form in the Syk tSH2 *holo*-state (Fig. 6*E*). This indicates that the final transition to the *holo*-state does not require the allosteric network to assemble. To test that, we determine the steady-state binding of ITAM-Y2P-ζ1 to Syk tSH2 mutant, ^tSH2^F120A and ^tSH2^W168C, respectively (Figs 5*A*, 6, *F* and *G*). None of the mutants altered the ITAM-Y2P binding, implying that the allosteric network is nonfunctional in Syk.

To explain if the absence of a penalty in Syk could lead to the hyperbolic binding in steady-state (Fig. 6*A*) and one-step binding kinetics (Fig. 6, *B* and *C*), we removed the kinetic penalty in all pathways ($w_{i}=1$) in the model described before (compare Figs. 2*B* and 6*H*). We further assumed that the closed conformation in the *apo*-state (the central species in the network, Fig. 6*I*) is short-lived. The experimental data of ITAM-Y2P binding to Syk matched with the modified model (Fig. 6*I*), producing a single exponential decay (Compare Figs. 6*J* and 2*F*). Therefore, the same kinetic model used for ZAP-70, in principle, also explains ligand binding in Syk if we consider independent binding in two SH2 domains (20, 34). This suggests that Syk tSH2 may have little or no penalty, explaining the lack of specificity for ITAM sequence (36, 51). Indeed, titration with ITAM-YP showed that Syk tSH2 domain binds with relatively stronger affinity (*K*_*d*_ = 27.7 ± 2.3 nM) than ITAM-Y2P (*K*_*d*_ = 65 ± 12 nM) (Figs. 6*A*, S7, *A* and *B*), suggesting the presence of negative cooperativity, as reported previously (52). We conclude the thermodynamic brake is unique to ZAP-70, which may have coevolved in the adaptive immune cells during the divergence of the BCR-TCR-MHC like the immune system in the jawed fish.

## Conclusions

In summary, we have presented a unified kinetic model that explains the ZAP-70 and Syk tSH2 domain recruitment to the ITAM motifs at the membrane. Our model explains the recently observed closed conformation of an isolated *apo*-tSH2 domain structure of Syk (30). Due to the absence of a penalty step, the Syk tSH2 domains could spontaneously adopt a closed conformation like in the *holo*-state, allowing the tSH2 domain to bind a wide range of ITAM-Y2P sequences with less selectivity. Absence of thermodynamic penalty facilitates binding of ITAM-Y2P even at lower concentrations observed under the basal level in B cells.

Our model suggests that incorporating a penalty step (thermodynamic brake) during the final transitioning to the holo-state (Fig. 4*M*) is enough to explain the biphasic ITAM-Y2P binding and selectivity in the ZAP-70 tSH2 domain. Alternatively, a bivalent avidity model comprised of two low-affinity interactions (*K*_d_ in μM range) could generate a higher affinity binding, which may explain a biphasic response (53). However, we could not detect two low-affinity binding events, in contrast our data (Fig. 3*C*) suggest that the C-SH2 and N-SH2 PBP of ZAP-70 binds sequentially with an nM and μM *K*_d_, respectively. Thus, we prefer the cooperative model comprised of a penalty step (Fig. 2*A*) to explain the biphasic ligand binding to ZAP-70.

We propose that the thermodynamic brake is an essential component in the kinetic proofreading mechanism of ZAP-70 regulation. The residues constituting the thermodynamic brake are conserved in all ZAP-70 kinases in the vertebrates, which coincides with the evolution of the humoral and cell-mediated immune system in jawed fish. Several rate-limiting steps kinetically regulate the initiation of the ZAP-70-dependent TCR signaling, including recruitment of coreceptor molecules coupled to Lck, dwell-time of ZAP-70, and phosphorylation of Y132 in LAT by ZAP-70 (49, 54, 55). In the T cell quiescence, the thermodynamic brake shifts the conformational equilibrium of the *apo*-tSH2 domain of ZAP-70 toward the open state. The additional energy barrier further stabilizes the compact inactive conformation of the kinase domain, which may shorten ZAP-70 dwell time at the TCR, causing reduced basal activation. The penalty step introduces a delay between the encounter complex formation and subsequent transition to the *holo*-tSH2 structure required for the activation of ZAP-70, which most likely explains delayed ZAP-70 recruitment to the TCR microcluster (32) and subsequent Ca^2+^ release in T cell (12). The thermodynamic brake may provide an added layer of regulation in the kinetic proofreading, fundamentally differentiating TCR from BCR response. However, we noted that ZAP-70 recruitment to the TCR microcluster is significantly slower than the $k_{obs}^{slow}$ measured in our *in vitro* experiments. Under the basal condition, the CD 3ξ chain is embedded into the plasma membrane (56, 57) and the rate of phosphorylation of the ITAM motif is regulated by a delicate balance between the activation of kinases, phosphatase, and the intracellular ion concentration (32, 58). A complete understanding of how the proposed thermodynamic brake modulates the ZAP-70 dwell time at the membrane needs future investigation.

The multiplicity of CD3 ITAM motifs provides tuneable amplification of downstream signaling (14), regulates T cell proliferation, secures effective negative selection, and prevents autoimmunity (59, 60, 61). The CD3- ζ is comprised of three ITAM motifs (numbered 1–3), and rest of the CD3 chains (γ, δ, and ε) contain one ITAM motif each. The tSH2 domain of ZAP-70 displays hierarchical preference in binding to the respective doubly phosphorylated ITAM motifs (41). The functional significance of sequence diversity of ITAM motifs and the preferential binding to the tSH2 domain is an open question. The ITAM sequence diversity in CD3 chains is required to regulate thymocyte selection during the T cell maturation (62, 63, 64). Apart from its role in transducing TCR signaling in response to antigen binding, ZAP-70 is essential for pre-T cell signaling during early thymocyte development (65). A temporal separation between the Syk and ZAP-70 activity is crucial for the thymocyte selection during the double-negative stage (DN3) to the double-positive stage (66, 67). We speculate that the ITAM multiplicity and the differential binding to the tSH2 domain of Syk and ZAP-70, respectively, could constitute an additional proofreading step that may help discriminate signals during thymocytes' negative and positive selection.

## Experimental procedures

### Constructs

ZAP-70 tSH2 WT (1–256), cloned in pSKB2 vector, was gifted from Prof. John Kuriyan, UC Berkeley. Syk tSH2 (7–263), cloned in pGEX6P1 vector, was gifted from Bruce Mayer (Addgene plasmid (Syk(NC)-SH2) #46521;). PCR-based site-directed mutagenesis was done in the tSH2 background (R39A, R43P, F117A, W165C, R192A).

### tSH2 domain expression and purification

The tSH2 domain of ZAP-70 and Syk was expressed and purified as explained previously (33). Briefly, the tSH2 domain of ZAP-70 was expressed in *E. coli* -BL21(DE3) cells by IPTG induction and purified using Ni-NTA column. The eluted protein from the Ni-NTA column was further purified using a Q-column, followed by gel filtration chromatography. The purified tSH2 domain was buffer exchanged to 20 mM Tris–Cl, pH 8.0, 150 mM NaCl, 5 mM β-mercaptoethanol, and 5% glycerol and stored at -80 °C. The Syk tSH2 domain was expressed as GST fusion tag (68, 69) in *E. coli*-BL21(DE3) cells by IPTG induction. The protein was purified using GSH column by eluting with reduced glutathione–containing buffer (50 mM Tris–cl pH 8, 10 mM reduced glutathione, 10% glycerol). The GST tag was removed by digesting the protein with Prescission protease for overnight at 4 °C. The digested sample was run over GSH column, followed by gel filtration chromatography for further purification. The purified constructs were stored in (20 mM Tris–Cl, pH8.0, 150 mM NaCl, 5% glycerol, and 5 mM β-mercaptoethanol) at −80 °C. The quality of the sample was determined by mass spectroscopy.

### Mass spectroscopy

The absolute mass of the tSH2 domains of ZAP-70 and Syk was measured using the Ultraflextreme MALDI-TOF/TOF mass spectrometer. The purified protein was buffer exchanged to 10 mM ammonium bicarbonate using a HiTrap Desalting Column (Cytiva). Then, 20 μM of the protein was mixed with the sinapinic acid matrix in a 1:1 ratio, loaded on the 384 well target plate, and dried for 1 h. The samples were pulsed with the smartbeam-II laser, and the data were collected in linear mode.

### Steady-state fluorescence experiments

Interaction between tSH2 domain and ITAM-Y2P in steady-state were measured at 25 °C by following the change in intrinsic tryptophan fluorescence upon ITAM-Y2P binding by using PTI spectrofluorometer (33). All the ITAM-Y2P peptides were purchased from Biotechdesk and GMRF group labs. 0.5 μM of tSH2 domain was titrated with various concentration of ITAM-Y2P peptide. The tryptophan fluorescence was recorded at λ_ex_ 295 nm and the emission was recorded from 300 nm to 400 nm. The dissociation constant ($K_{d}$) was derived by fitting the normalized intensity (F_0_/F) *versus* ligand concentration using the following equation:

$$\frac{F_{0}}{F}=B_{max}*X^{n_{H}}/\left({K_{d}}^{n_{H}}+X^{n_{H}}\right)$$

where F_0_ and F is fluorescence intensity in absence and presence of ligand, respectively. B_max_ is the maximum binding, and nH is the Hill coefficient. The Hill coefficient (nH) was also determined from Hill plot (70). The change in Gibb’s free energy ($\Delta G_{binding}$) for each ligand binding step were calculated using ΔG_binding_ = -RTlnK, where K=1/ $K_{d}$. The total change in Gibb’s free energy $\Delta G_{binding}$ was calculated by adding the respective $\Delta G_{binding}$ at each step for respective tSH2 domain: ITAM-Y2P interaction. $\Delta \Delta G_{binding}$ was calculated by subtracting $\Delta G_{binding}^{total}$ for the WT tSH2 domain: ITAM-Y2P-ζ1 from the indicated tSH2:ITAM-Y2P interactions (Fig. 4*L*).

### Pre-steady state kinetics

The binding kinetics between tSH2 domain and ITAM-Y2P ligands were measured by following change in tryptophan fluorescence using a stopped-flow fluorimeter (SFM2000 BioLogic Spectrophotometer). The change in fluorescence intensity was measured at λ_ex_ 290 nm, λ_em_ 350 nm, and 10 °C (71). Hundred nanomolar of protein (tSH2 domain) in 20 mM Tris (pH 8.0), 150 mM NaCl, 5% glycerol, 5 mM β-mercaptoethanol, and 30 μM of ITAM-Y2P in same buffer, were mixed using syringe. Each transient was measured over 200s (for slow kinetics) or 1s (for fast kinetics), interval with 1500 and 101 time points, respectively. For each sample, a blank dataset was recorded by measuring the change in intensity for protein only. Each data set was normalized against the maximum intensity observed for the respective sample at time 0. The normalized sample was further corrected by blank subtraction. The observed rate (*k*_*obs*_) was derived by fitting the change in intensity with respect to time by fitting to association kinetics equation implemented in Prism.

$$Y=Y_{0}+\left(Y_{max}-Y_{0}\right)\left(1-e^{-k20t}\right)$$

where, $Y_{0}$ is the intensity at time 0, $Y_{max}$ is the maximum intensity, k is rate constant.

### Isothermal titration calorimetry

ITC studies were carried out using Malvern-PEAQ_ITC at 20 °C. The WT tSH2 domain at a concentration of 20 μM was titrated to different ITAM-Y2P constructs in glycerol-free Tris buffer (20 mM Tris–Cl, pH8.0, 150 mM NaCl, 5 mM β-mercaptoethanol). The $K_{d}$, ΔH, and ΔS were fitted into two-site sequential fit using ORIGIN as described previously (33). In the ITC experiments with C-SH2 domain mutant (R190A), the protein was buffer exchanged to 20 mM Hepes pH 8.2, 150 mM NaCl, and 5 mM β-mercaptoethanol. Titrations were carried out with 300 μM of ^ITAM-Y2P-ζ1^EI3A and ITAM-Y2P-ζ3 peptides and the data was fitted to one-site binding model to determine the $K_{d}$, ΔH, and ΔS.

### Phylogenetic analysis

The full-length protein sequences for Syk related kinases, Syk and ZAP-70, were acquired using multiple blast searches in the Uniprot database and PSI blast in NCBI. *Homo sapiens* Syk and ZAP-70 were separately used as query sequences against invertebrates and vertebrates. (Tables S1 and S2) (72, 73, 74). The secondary structures of proteins were verified using the PROSITE Expasy(Fig. S5) (75). Only the proteins with tSH2 domains connected by a linker, which is connected to a kinase domain as seen in the *H. sapiens* ZAP-70 with comparable interdomain A and B lengths were selected for sequence analysis. TK4, a Syk family kinase found in Platyhelminthes, was not considered for analysis due to considerably longer interdomain B (594 residues) than Syk kinase in *H. sapiens* (110 residues). Also, HTK16 in *Hydra Vulgaris* and SHARK in *Drosophila* is not included in our analysis, since they possess Ankyrin repeats between the two SH2 domains. The protein tyrosine kinase in *Eptatretus burgeri* (jawless fish) was also removed as it comprises PH, Btk-type zinc finger, SH2, SH3, and Kinase domains. A total of 148 sequences from 82 organisms were selected, and accession numbers are summarized in Tables S1 and S2. All the sequences were aligned, and a phylogenetic tree was constructed using MEGAX with the maximum likelihood method and bootstrapped 1000 times (76, 77, 78, 79). The phylogenetic tree was visualized using iTOL (80). The WEB LOGO was used to visualize allosteric network residue conservation in Syk and ZAP-70 (81, 82).

### Mathematical analysis of kinetic models

Using laws of mass action, we derived the following set of ordinary differential equations (ODEs) from the kinetic full model shown in Figure 2*B*

(S1) $$\begin{array}{l} \frac{dR_{open}^{00}}{\mathrm{dt}}=k_{b}R_{open}^{01}+k_{b3}R_{closed}^{10}+k_{open}^{\prime }R_{closed}^{00}-\left(1+w_{3}\right)k_{f}\text{L}R_{open}^{00}-k_{close}^{\prime }R_{open}^{00}\\ \frac{dR_{closed}^{00}}{\mathrm{dt}}=k_{close}^{\prime }R_{open}^{00}+k_{1}^{-}R_{closed}^{10}+k_{2}^{-}R_{closed}^{01}+k_{3}^{-}R_{closed}^{11}-k_{open}^{\prime }R_{closed}^{00}-\left(k_{1}^{+}+k_{2}^{+}\right)\text{L}R_{closed}^{00}-k_{3}^{+}L^{2}R_{closed}^{00}\\ \frac{dR_{open}^{01}}{\mathrm{dt}}=k_{f}\text{L}R_{open}^{00}+k_{open}R_{closed}^{01}-\left(k_{b}+k_{close}\right)R_{open}^{01}\\ \frac{dR_{closed}^{01}}{\mathrm{dt}}=k_{close}R_{open}^{01}+k_{b1}R_{closed}^{11}+k_{2}^{+}\text{L}R_{closed}^{00}-k_{open}R_{closed}^{01}-k_{2}^{-}R_{closed}^{01}-w_{1}k_{f}\text{L}R_{closed}^{01}\\ \frac{dR_{closed}^{10}}{\mathrm{dt}}=w_{3}k_{f}\text{L}R_{open}^{00}+k_{b2}R_{closed}^{11}+k_{1}^{+}\text{L}R_{closed}^{00}-k_{b3}R_{closed}^{10}-k_{1}^{-}R_{closed}^{10}-w_{2}k_{f}\text{L}R_{closed}^{10}\\ \frac{dR_{closed}^{11}}{\mathrm{dt}}=w_{1}k_{f}\text{L}R_{closed}^{01}+w_{2}k_{f}\text{L}R_{closed}^{10}+k_{3}^{+}L^{2}R_{closed}^{00}-\left(k_{b1}+k_{b2}\right)R_{closed}^{11}-k_{3}^{-}R_{closed}^{11} \end{array}\}$$

All the notations for each chemical species involved in our model are summarized in Figure 2*A*, and L denotes the concentration of the ligand. In our model, as described previously (33), the ligand first binds to the C-SH2 PBP converting the *apo-*state ($R_{open}^{00}$) to the encounter complex, $R_{open}^{01}$. We assume that the encounter complex undergoes ligand-independent conformational change that brings the two SH2 domains close to each other forming the partially bound closed intermediate ($R_{closed}^{01}$). We also assumed two other closed intermediates, $R_{closed}^{00}$ and $R_{closed}^{10}:$ (i) the *apo*-state ($R_{open}^{00}$) may directly undergo conformational transition to an unligated closed intermediate ($R_{closed}^{00}$) and (ii) there exists another partially bound closed state, $R_{closed}^{10}$ (N-SH2 PBP is bound but C-SH2 PBP is free), which may arise from $R_{closed}^{00}$ , $R_{open}^{00},$ or $R_{closed}^{11}$ (see Fig. 2*B*). Since there is allosteric interaction between the two SH2 domains, we considered kinetic penalties (*i.e.*, reduced forward kinetics compared to the backward) in the transitions from the partially bound states ($R_{closed}^{10}$ or $R_{closed}^{01}$) to the final *holo*-state ($R_{closed}^{11}$). These penalties were introduced mathematically by the factors $w_{1}$ and $w_{2}$ that were assumed to have values less than 1. The penalty-inducing steps are mainly the following: (i) from $R_{closed}^{01}$ to $R_{closed}^{11}$ with a dissociation constant $K_{d2}$ ($K_{d2}=k_{b1}/w_{1}k_{f}$) and (ii) from $R_{closed}^{10}$ to $R_{closed}^{11}$ with a dissociation constant $K_{d1}^{*}$ ($K_{d1}^{*}=k_{b2}/w_{2}k_{f}$). Experiments suggest that $K_{d2}$ and $K_{d1}^{*}$ are around 3 to 12 μM and 45 to 140 nM, respectively, while the dissociation constant for the transition from $R_{open}^{00}$ to $R_{open}^{01}$ ($K_{d1}$) is around 3 to 10 nM (see Fig. 1*E* and Table S3) (33). Therefore, we first fixed these parameter values in our simulations as $K_{d1}=2$ nM, $K_{d2}=10$ μM, and $K_{d1}^{*}=100$ nM and then varied any one of the $K_{d}$ values to determine its effect.

In the ODEs (Equation S1), kinetic rates are, in general, denoted by $k_{i}$ , where *i* symbolically represents the respective forward or backward transitions for each species (see Fig. 2*B*). To form the ODEs based on the mass-action principle, we assumed a single binding event at any of the two sites (N-SH2 or C-SH2 PBP) as first order kinetics (*i.e.*, binding rate is proportional to L) and double binding events as second order kinetics (*i.e.*, proportional to L^2^). It is to be noted that our kinetic model is mainly based on the allosteric interaction of ZAP-70 tSH2 domain and does not contain any feedback (43, 44).

Following Sevlever *et al. 2020* (42), the ‘bound fraction’ is defined as the proportion of three species, two partially bound closed intermediates ($R_{closed}^{01}$, $R_{closed}^{10}$) and the closed *holo*-state ($R_{closed}^{11}$). The formula for the bound fraction is given by

(S2) $${\text{Φ}}_{b}=\frac{\left(\frac{1}{2}R_{closed}^{01}+\frac{1}{2}R_{closed}^{10}+R_{closed}^{11}\right)}{R_{total}}$$

where $R_{total}=\left(R_{open}^{00}+R_{\text{open}}^{01}+R_{\text{closed}}^{01}+R_{\text{closed}}^{10}+R_{\text{closed}}^{00}+R_{\text{closed}}^{11}\right)$. In the Equation S2, the ½ factors in the numerator (associated with $R_{\text{closed}}^{01}$ and $R_{\text{closed}}^{10}$ ) account the fact that only one of the two PBPs are occupied by the ligand. Conversely, the ‘unbound fraction’ is given by ${\text{Φ}}_{ub}=\left(1-{\text{Φ}}_{b}\right)$.

We solved the above set of ODEs (Equation S1) numerically in Mathematica (using Parametric NDSOLVE) to obtain the concentrations for each species. We assumed that the steady-state has reached when all concentrations were constant at a large time (we took numerical data at t >> 5 h). From steady-state concentrations, we calculated the bound fraction using the Equation S2 and then plotted them against the ligand concentrations (in Fig. 2, *C* and *F*). The initial concentrations were as follows: $R_{open}^{00}$ = 500 nM and $R_{closed}^{00}=R_{open}^{01}=R_{closed}^{01}=R_{closed}^{10}=R_{closed}^{11}=0$, at t = 0.

To observe the kinetic behavior, we numerically solved the same set of ODEs (Equation S1) under the saturating ligand concentration (L >> $R_{open}^{00}$). To check consistency, we performed multiple simulations of the kinetic profiles with different initial ligand concentrations (L), 5 to 10 times higher than the receptor concentration ($R_{open}^{00}$). We mostly used initial concentrations of $R_{open}^{00}$ = 500 nM and L = 10 μM (Figs. 2, *G*, *H*, S2, *A* and *B*) as representative plots. However, we used $\text{initial concentrations of }R_{open}^{00}$ = 100 nM and L = 0.5 μM in Fig. 2, *D* and *E* for visual clarity since the curves are well separated from each other and the trend of the $K_{d1}$ variation can be clearly seen. Nevertheless, in Fig S2, *A* and *B*, we have shown that choosing the initial concentration of $R_{open}^{00}$ = 500 nM and L = 10 μM produces the same effect of $K_{d1}$ variation. All other parameter values used to produce the kinetic profiles are summarized in Table S4.

To match the theoretical bound fraction with the experimental data (Fig. 2*I*), we calculated the bound fraction from the experimental data as below:

(S3) $${\text{Φ}}_{b}\;\left(\text{experimental}\right)\;=\;\frac{\left(F_{max}-F\right)}{\left(F_{max}-F_{min}\right)}$$

where F is the fluorescence intensity and $F_{max}$ and $F_{min}$ are the maximum and minimum intensities, respectively.

### Modified model for tSH2: ITAM-YP-ζ1 and ^tSH2^R39A: ITAM-Y2P-ζ1 interaction

We introduced a modified model (see Fig. 3*F*) to explain the binding kinetics of tSH2: ITAM-YP-ζ1 and ^tSH2^R39A: ITAM-Y2P-ζ1 in the steady-state. For both the tSH2: ITAM-YP-ζ1 and ^tSH2^R39A: ITAM-Y2P-ζ1 interactions, the N-SH2 binding is almost absent, and hence, other intermediates ($R_{closed}^{00}\text{ and }R_{closed}^{10}$) and the final *holo*-state ($R_{closed}^{11}$) cannot be formed. We, therefore, neglect these species ($R_{closed}^{00},R_{closed}^{10}$, and $R_{closed}^{11}$) in the full kinetic model (Fig. 2*B*). Accordingly, we set $w_{1}=w_{2}=w_{3}=0$ and {$k_{b1},$ $k_{b2},$ $k_{b3},k_{1}^{+},k_{1}^{-},k_{2}^{+},k_{2}^{-},k_{3}^{+},k_{3}^{-},$ $k_{close}^{\prime }$ , $k_{open}^{\prime }$ } ∼ 0 (*i.e.*, negligible) in our model.

In this case, since only the C-SH2 PBP can be occupied, the bound fraction would be ${\text{Φ}}_{b}=\left(\frac{R^{01}}{R_{total}}\right)$. The bound fraction for both tSH2: ITAM-YP-ζ1 and ^tSH2^R39A: ITAM-Y2P-ζ1 are plotted in Figure 3*G* and the kinetic profiles of the unbound fraction are shown in Figure 3*H*. The corresponding parameter values used to match the theoretical bound fraction with the experimental data are mentioned in Table S6. According to our experimental data, C-SH2 binding affinity are similar for tSH2: ITAM-YP-ζ1 and ^tSH2^R39A: ITAM-Y2P-ζ1 ($K_{d1}$ = 4 nM and 8 nM, respectively; Fig. S3*B*). However, our fitted parameters suggest that the rate of transition of closed to open conformational change ($k_{open}$ in Fig. 3*F*) are different for tSH2: ITAM-YP-ζ1 and ^tSH2^R39A: ITAM-Y2P-ζ1 (Table S6).

### Modified model for ITAM-Y2P and Syk tSH2 domain interaction

Binding of ITAM-Y2P-ζ1 and ITAM-Y2P-ζ3 with Syk were also explained by a modified model (Fig. 6*H*). We solved the same set of ODEs (Equation S1) with the conditions:

$w_{1}=w_{2}=w_{3}=1,k_{b1}=k_{b2}=k_{b3}=k_{b}\left(\text{i}.\text{e}.,\text{no}\;\text{kinetic}\;\text{penalty}\right)$

and

$\{k_{1}^{+},k_{1}^{-},k_{2}^{+},k_{2}^{-},k_{3}^{+},k_{3}^{-},$$k_{close}^{\prime }$, $k_{open}^{\prime }$ }∼ 0 (*i.e.* negligible).

The bound fraction in the steady-state for three different $K_{d}$ values were plotted against the ligand concentration in Figure 6*I* and kinetic profiles of the unbound fraction are shown in Figure 6*J*. Parameters corresponding to these plots are shown in Table S7. According to the experiments, Syk showed similar $K_{d}$ values (around 65–70 nM) for ITAM-Y2P-ζ1 and ITAM-Y2P-ζ3 peptides. Hence, we varied $K_{d}$ values ranging from 20 nM to 100 nM in our simulations to produce a quantitative match between the theoretical and the experimental data.

### Reduced kinetic model

Our experimental data (Figs. 3, *C*–*E* and S3, *A*, *B*, and *G*) and our previous results (33) suggest that the encounter complex ($R_{open}^{01}$) is indispensable in transitioning to the *holo*-complex ($R_{closed}^{11}$). This observation motivated us to build a simpler reduced model (Fig. 3*K*) from the full complex model (Fig. 2*B*). Specifically, we considered two simplifications: (i) we eliminated the direct transition from $R_{open}^{00}$ to $R_{closed}^{10}$ , because $R_{closed}^{10}$ is unlikely to form in the substoichiometric ligand concentration (Fig. S3*G*) (33) and the formation of $R_{closed}^{10}$ is conditional on the formation of the encounter complex ($R_{open}^{01}$); (ii) we removed the possibility of forming the ligand-independent closed intermediate ($R_{closed}^{00}$) since our previous acrylamide quenching of tryptophan fluorescence (indicated by Stern-Volmer constant, *K*_*SV*_) revealed that the formation of the encounter complex is prerequisite for bringing the two SH2-domains close to each other (33).

Similar to the full model in Figure 2*B*, we have derived the ODEs for the reduced model shown in Figure 3*K*. The ODEs are as follows:

(S4) $$\begin{array}{l} \frac{dR_{open}^{00}}{dt}=k_{b}R_{open}^{01}-k_{f}\text{L}R_{open}^{00}\\ \frac{dR_{open}^{01}}{dt}=k_{f}\text{L}R_{open}^{00}+k_{-}R_{closed}^{01/10}+k_{b}R_{closed}^{11}-\left(k_{b}+k_{+}+w_{1}k_{f}\text{L}\right)R_{open}^{01}\\ \frac{dR_{closed}^{01/10}}{dt}=k_{+}R_{open}^{01}+k_{b}R_{closed}^{11}-\left(k_{-}+w_{2}k_{f}\text{L}\right)R_{closed}^{01/10}\\ \frac{dR_{closed}^{11}}{dt}=w_{1}k_{f}\text{L}R_{open}^{01}+w_{2}k_{f}\text{L}R_{closed}^{01/10}-2k_{b}R_{closed}^{11} \end{array}\}$$

Here, $R_{closed}^{01/10}$ denotes the concentration of partially bound closed intermediates (*i.e.*, either N-SH2 PBP or C-SH2 PBP is bound to the ligand). Other chemical species are as mentioned in Figure 2*A*. Similar to the full model, the transitions between open and closed conformations (*via* rates $k_{+}$ and $k_{-}$) are ligand independent.

Solving the above set of ODEs in the steady-state (Equation S4), we derived exact expressions for the concentrations as follows

(S5) $$\begin{array}{l} R_{open}^{01}=\left(\frac{L}{K_{d1}}\right)\times R_{open}^{00}\\ R_{closed}^{01/10}=\left(\frac{2k_{+}+w_{1}k_{f}L}{2k_{-}+w_{2}k_{f}L}\right)\times \left(\frac{L}{K_{d1}}\right)\times R_{open}^{00}\\ R_{closed}^{11}=\left(\frac{w_{1}k_{-}+w_{2}\left(k_{+}+w_{1}k_{f}L\right)}{2k_{-}+w_{2}k_{f}L}\right)\times {\left(\frac{L}{K_{d1}}\right)}^{2}\times R_{open}^{00} \end{array}\}$$

where, $K_{d1}$ = $\frac{k_{b}}{k_{f}}$

We used the same parameter values for $k_{f}$, $k_{b}$, $w_{1}$, $w_{2}$ as in Table S4, while we took $k_{+}$ = 0.00007/s, $k_{-}$ =0.0007/s $\left(\frac{k_{-}}{k_{+}}=10\right)$. From the above expressions (Equation S5), we calculated the bound-fraction and the unbound-fraction as below

$${\text{Φ}}_{b}=\frac{\left(\frac{1}{2}\;R_{open}^{01}+\;\frac{1}{\;2}\;R_{closed}^{01/10}+R_{closed}^{11}\right)}{\left(R_{open}^{00}+\;R_{open}^{01}+R_{closed}^{01/10}+R_{closed}^{11}\right)}\;\text{and}\;{\text{Φ}}_{ub}=1-{\text{Φ}}_{b}$$

The bound and unbound fractions for the reduced model are plotted in Fig. S3, *C*–*F*, which qualitatively show the same results as in the full model (compare Figs. 2, *C*–*H*, and S3, *C*–*F*).

Mathematica Codes: all codes are publicly available online at the following link https://github.com/arnabroy97/ODE_calculations

## Data availability

All the relevant data are contained within this article and in the supporting information.

## Supporting information

The supporting information contains Figures S1–S7 and Table S1–S7.

## Conflict of interest

The authors declare that they have no conflict of interest with the contents of this article.
