# Supplementary material for: An evolutionary divergent thermodynamic brake in ZAP-70 fine-tunes the kinetic proofreading in T cells
Source: J Biol Chem. 2022 Aug 13;298(10):102376. doi: 10.1016/j.jbc.2022.102376 (PMC9486129; doi:10.1016/j.jbc.2022.102376)
Supplement: Supplemental information [file mmc1.pdf]

SUPPORTING INFORMATION

An evolutionary divergent thermodynamic brake in ZAP-70 fine-tunes the kinetic proofreading in T cells

Kaustav Gangopadhyay[a]+, Arnab Roy[a]+, Athira C. Chandradasan[a], Swarnendu Roy[a], Olivia Debnath[a], Soumee SenGupta[a], Subhankar Chowdhury[a], Dipjyoti Das[a]* and Rahul Das[a],[b]*

[a] Department of Biological Sciences, Indian Institute of Science Education and Research Kolkata, Mohanpur campus, Mohanpur-741246, India

[b] Centre for Advanced Functional Materials, Indian Institute of Science Education and Research Kolkata, Mohanpur campus, Mohanpur-741246, India

[+] These authors made equal contributions.

Manuscript

Corresponding authors

Rahul Das: rahul.das@iiserkol.ac.in

Dipjyoti Das: dipjyoti.das@iiserkol.ac.in

Running Title: Thermodynamic brake in ZAP-70

Keywords: Kinase, Cell signaling, Kinetic model, T cell, Proofreading, ZAP-70

Supplementary Tables and Figures

Table S1: Accession number of Syk and Syk Related Kinases of different organisms

Sl. no	Accession number	Gene	Species	Phylum	Class	Domains
1	Q9Y1X9	EfPTK151	*Ephydatia fluviatilis*	Porifera	Demospongiae	2 SH2, 1 kinase
2	A0A1X7V2S5	100640238	*Amphimedon queenslandica*	Porifera	Demospongiae	2 SH2, 1 kinase
3	O77440	HTK98	*Hydra vulgaris*	Cnidaria	Hydrozoa	2 SH2, 1 kinase
4	A0A2B4SFM1	SYK	*Stylophora pistillata*	Cnidaria	Anthozoa	2 SH2, 1 Kinase
5	A0A6P8ILN3	LOC116302664	*Actinia tenebrosa*	Cnidaria	Anthozoa	2 SH2, 1 Kinase
6	XP_020903367.1	SYK IsoformX1	*Exaiptasia diaphana*	Cnidaria	Anthozoa	2 SH2, 1 Kinase
7	XP_032226357.1	SYK IsoformX1	*Nematostella vectensis*	Cnidaria	Anthozoa	2 SH2, 1 Kinase
8	XP_028390911.1	SYK IsoformX1	*Dendronephthya gigantea*	Cnidaria	Anthozoa	2 SH2, 1 Kinase
9	XP_021340359.1	SYK-like	*Mizuhopecten yessoensis*	Mollusca	Bivalvia	2 SH2, 1 Kinase
10	CAG2209972.1	SYK	*Mytilus edulis*	Mollusca	Bivalvia	2 SH2, 1 Kinase
11	XP_033736612.1	SYK-like	*Pecten maximus*	Mollusca	Bivalvia	2 SH2, 1 Kinase
12	XP_041360389.1	SYK-like	*Gigantopelta aegis*	Mollusca	Gastropoda	2 SH2, 1 Kinase
13	XP_038067744.1	SYK IsoformX1	*Patiria miniata*	Echinodermata	Asteroidea	2 SH2, 1 Kinase
14	XP_030851825.1	SYK IsoformX1	*Strongylocentrotus purpuratus*	Echinodermata	Echinoidea	2 SH2, 1 Kinase
15	XP_032816776.1	SYK IsoformX1	*Petromyzon marinus*	Chordata	Agnatha	2 SH2, 1 Kinase
16	A0A0C5DLZ7	SYK	*Lethenteron camtschaticum*	Chordata	Agnatha	2 SH2, 1 Kinase
17	A0A4W3IP53	SYK	*Callorhinchus milii*	Chordata	Chondrichthyes	2 SH2, 1 Kinase
18	A0A401SZZ3	chiPu_0014454	*Chiloscyllium punctatum*	Chordata	Chondrichthyes	2 SH2, 1 Kinase
19	XP_041042106.1	SYK IsoformX1	*Carcharodon carcharias*	Chordata	Chondrichthyes	2 SH2, 1 Kinase
20	XP_032873635.1	SYK IsoformX1	*Amblyraja radiata*	Chordata	Chondrichthyes	2 SH2, 1 Kinase
21	XP_038660658.1	SYK IsoformX1	*Scyliorhinus canicula*	Chordata	Chondrichthyes	2 SH2, 1 Kinase
22	XP_008323672.1	SYK IsoformX1	*Cynoglossus semilaevis*	Chordata	Osteichthyes	2 SH2, 1 Kinase
23	A0A672HYJ7	SYK	*Salarias fasciatus*	Chordata	Osteichthyes	2 SH2, 1 Kinase
24	XP_014191223.1	SYK IsoformX1	*Haplochromis burtoni*	Chordata	Osteichthyes	2 SH2, 1 Kinase
25	XP_015191917.1	SYK IsoformX1	*Lepisosteus oculatus*	Chordata	Osteichthyes	2 SH2, 1 Kinase
26	H3ATL1	SYK	*Latimeria chalumnae*	Chordata	Osteichthyes	2 SH2, 1 Kinase
27	XP_026152626.1	SYK	*Mastacembelus armatus*	Chordata	Osteichthyes	2 SH2, 1 Kinase
28	RXM97385.1	SYK	*Acipenser ruthenus*	Chordata	Osteichthyes	2 SH2, 1 Kinase
29	NP_998008.2	SYK	*Danio rerio*	Chordata	Osteichthyes	2 SH2, 1 Kinase
30	A0A672MPJ9	SYK	*Sinocyclocheilus grahami*	Chordata	Osteichthyes	2 SH2, 1 Kinase
31	KAG9263497.1	SYK	*Astyanax mexicanus*	Chordata	Osteichthyes	2 SH2, 1 Kinase

32	XP_026798305.1	SYK	*Pangasianodon hypophthalmus*	Chordata	Osteichthyes	2 SH2, 1 Kinase
33	XP_012711475.1	SYK IsoformX1	*Fundulus heteroclitus*	Chordata	Osteichthyes	2 SH2, 1 Kinase
34	XP_004564629.1	SYK	*Maylandia zebra*	Chordata	Osteichthyes	2 SH2, 1 Kinase
35	XP_018600403.1	SYK IsoformX1	*Scleropages formosus*	Chordata	Osteichthyes	2 SH2, 1 Kinase
36	A0A1L8HYI7	SYK.L	*Xenopus laevis*	Chordata	Amphibia	2 SH2, 1 Kinase
37	F7DXJ8	SYK	*Xenopus tropicalis*	Chordata	Amphibia	2 SH2, 1 Kinase
38	A0A6P8PLK0	SYK	*Geotrypetes seraphini*	Chordata	Amphibia	2 SH2, 1 Kinase
39	A0A6P7X829	SYK	*Microcaecilia unicolor*	Chordata	Amphibia	2 SH2, 1 Kinase
40	XP_040272878.1	SYK IsoformX1	*Bufo bufo*	Chordata	Amphibia	2 SH2, 1 Kinase
41	XP_029474555.1	SYK IsoformX1	*Rhinatrema bivittatum*	Chordata	Amphibia	2 SH2, 1 Kinase
42	A0A6J0U1P1	SYK	*Pogona vitticeps*	Chordata	Reptile	2 SH2, 1 Kinase
43	XP_005307710.1	SYK IsoformX1	*Chrysemys picta bellii*	Chordata	Reptile	2 SH2, 1 Kinase
44	A0A402FJ14	parPi_0018852	*Paroedura picta*	Chordata	Reptile	2 SH2, 1 Kinase
45	XP_024070757.2	SYK IsoformX1	*Terrapene carolina triunguis*	Chordata	Reptile	2 SH2, 1 Kinase
46	A0A670JJY3	SYK	*Podarcis muralis*	Chordata	Reptile	2 SH2, 1 Kinase
47	A0A6I9XWC1	SYK	*Thamnophis sirtalis*	Chordata	Reptile	2 SH2, 1 Kinase
48	A0A674H2I4	SYK	*Taeniopygia guttata*	Chordata	Bird	2 SH2, 1 Kinase
49	A0A1V4J5M0	SYK	*Patagioenas fasciata monilis*	Chordata	Bird	2 SH2, 1 Kinase
50	A0A493TXF0	SYK	*Anas platyrhynchos*	Chordata	Bird	2 SH2, 1 Kinase
51	F1N9Y5	SYK	*Gallus gallus*	Chordata	Bird	2 SH2, 1 Kinase
52	A0A218UQF1	SYK	*Lonchura striata domestica*	Chordata	Bird	2 SH2, 1 Kinase
53	XP_026722123.1	SYK	*Athene cunicularia*	Chordata	Bird	2 SH2, 1 Kinase
54	XP_029861296.1	SYK IsoformX1	*Aquila chrysaetos chrysaetos*	Chordata	Bird	2 SH2, 1 Kinase
55	XP_040396840.1	SYK IsoformX1	*Cygnus olor*	Chordata	Bird	2 SH2, 1 Kinase
56	KAF1479555.1	SYK	*Megadyptes antipodes*	Chordata	Bird	2 SH2, 1 Kinase
57	U3JQ94	SYK	*Ficedula albicollis*	Chordata	Bird	2 SH2, 1 Kinase
58	A0A1U8BEZ1	SYK	*Mesocricetus auratus*	Chordata	Mammalia	2 SH2, 1 Kinase
59	A0A2K6F665	SYK	*Propithecus coquereli*	Chordata	Mammalia	2 SH2, 1 Kinase
60	P48025	SYK	*Mus musculus*	Chordata	Mammalia	2 SH2, 1 Kinase
61	A0A1S3AT02	SYK	*Erinaceus europaeus*	Chordata	Mammalia	2 SH2, 1 Kinase
62	A0A2I2UZ97	SYK	*Felis catus*	Chordata	Mammalia	2 SH2, 1 Kinase
63	W5PD78	SYK	*Ovis aries*	Chordata	Mammalia	2 SH2, 1 Kinase
64	A0A5F9CUA6	SYK	*Oryctolagus cuniculus*	Chordata	Mammalia	2 SH2, 1 Kinase
65	H0X5F7	SYK	*Otolemur garnettii*	Chordata	Mammalia	2 SH2, 1 Kinase
66	Q64725	SYK	*Rattus norvegicus*	Chordata	Mammalia	2 SH2, 1 Kinase
67	Q00655	SYK	*Sus scrofa*	Chordata	Mammalia	2 SH2, 1 Kinase

68	A0A2K6PUC6	SYK	*Rhinopithecus roxellana*	Chordata	Mammalia	2 SH2, 1 Kinase
69	U3BGC5	SYK	*Callithrix jacchus*	Chordata	Mammalia	2 SH2, 1 Kinase
70	F7GLV2	SYK	*Macaca mulatta*	Chordata	Mammalia	2 SH2, 1 Kinase
71	A0A2K5KN52	SYK	*Cercocebus atys*	Chordata	Mammalia	2 SH2, 1 Kinase
72	G1MGB0	SYK	*Ailuropoda melanoleuca*	Chordata	Mammalia	2 SH2, 1 Kinase
73	A0A096P223	SYK	*Papio anubis*	Chordata	Mammalia	2 SH2, 1 Kinase
74	XP_023483322.1	SYK IsoformX1	*Equus caballus*	Chordata	Mammalia	2 SH2, 1 Kinase
75	A0A0D9R4K5	SYK	*Chlorocebus sabaeus*	Chordata	Mammalia	2 SH2, 1 Kinase
76	XP_016816610.1	SYK	*Pan troglodytes*	Chordata	Mammalia	2 SH2, 1 Kinase
77	ERE77472.1	SYK	*Cricetulus griseus*	Chordata	Mammalia	2 SH2, 1 Kinase
78	A0A2K5PKV5	SYK	*Cebus imitator*	Chordata	Mammalia	2 SH2, 1 Kinase
79	A0A6I9L4D8	SYK	*Peromyscus maniculatus bairdii*	Chordata	Mammalia	2 SH2, 1 Kinase
80	G3R4D0	SYK	*Gorilla gorilla*	Chordata	Mammalia	2 SH2, 1 Kinase
81	G3T8M0	SYK	*Loxodonta africana*	Chordata	Mammalia	2 SH2, 1 Kinase
82	P43405	SYK	*Homo sapiens*	Chordata	Mammalia	2 SH2, 1 Kinase

Table S2: Accession number of ZAP-70 of different organisms

**Sl. no**	**Accession number**	**Gene**	**Species**	**Phylum**	**Class**	**Domains**
1	V9KJG4	ZAP-70	*Callorhinchus milii*	Chordata	Chondrichthyes	2 SH2, 1 Kinase
2	A0A401SFZ6	ChiPu_0007750	*Chiloscyllium punctatum*	Chordata	Chondrichthyes	2 SH2, 1 Kinase
3	XP_041061522.1	ZAP-70	*Carcharodon carcharias*	Chordata	Chondrichthyes	2 SH2, 1 Kinase
4	XP_032902714.1	ZAP-70	*Amblyraja radiata*	Chordata	Chondrichthyes	2 SH2, 1 Kinase
5	XP_038634070.1	ZAP-70 IsoformX1	*Scyliorhinus canicula*	Chordata	Chondrichthyes	2 SH2, 1 Kinase
6	XP_016896784.1	ZAP-70	*Cynoglossus semilaevis*	Chordata	Osteichthyes	2 SH2, 1 Kinase
7	A0A672JN05	LOC115382146	*Salarias fasciatus*	Chordata	Osteichthyes	2 SH2, 1 Kinase
8	XP_005938595.1	ZAP-70	*Haplochromis burtoni*	Chordata	Osteichthyes	2 SH2, 1 Kinase
9	XP_015221298.1	ZAP-70	*Lepisosteus oculatus*	Chordata	Osteichthyes	2 SH2, 1 Kinase
10	M3XJU1	ZAP-70	*Latimeria chalumnae*	Chordata	Osteichthyes	2 SH2, 1 Kinase
11	XP_026179399.1	ZAP-70	*Mastacembelus armatus*	Chordata	Osteichthyes	2 SH2, 1 Kinase
12	A0A662YJN3	EOD39_15354	*Acipenser ruthenus*	Chordata	Osteichthyes	2 SH2, 1 Kinase
13	NP_001018425.1	ZAP-70	*Danio rerio*	Chordata	Osteichthyes	2 SH2, 1 Kinase
14	A0A672LYH7	LOC107548917	*Sinocyclocheilus grahami*	Chordata	Osteichthyes	2 SH2, 1 Kinase

15	KAG9270088.1	ZAP-70	*Astyanax mexicanus*	Chordata	Osteichthyes	2 SH2, 1 Kinase
16	XP_026783834.1	ZAP-70	*Pangasianodon hypophthalmus*	Chordata	Osteichthyes	2 SH2, 1 Kinase
17	XP_012736872.2	ZAP-70	*Fundulus heteroclitus*	Chordata	Osteichthyes	2 SH2, 1 Kinase
18	XP_004553860.2	ZAP-70	*Maylandia zebra*	Chordata	Osteichthyes	2 SH2, 1 Kinase
19	XP_018583813.1	ZAP-70	*Scleropages formosus*	Chordata	Osteichthyes	2 SH2, 1 Kinase
20	Q6DCV6	ZAP-70.L	*Xenopus laevis*	Chordata	Amphibia	2 SH2, 1 Kinase
21	Q6DF54	ZAP-70	*Xenopus tropicalis*	Chordata	Amphibia	2 SH2, 1 Kinase
22	A0A6P8S0Q1	LOC117365392	*Geotrypetes seraphini*	Chordata	Amphibia	2 SH2, 1 Kinase
23	A0A6P7Z494	LOC115479942	*Microcaecilia unicolor*	Chordata	Amphibia	2 SH2, 1 Kinase
24	XP_040273715.1	ZAP-70 isoformX2	*Bufo bufo*	Chordata	Amphibia	2 SH2, 1 Kinase
25	XP_029466798.1	ZAP-70 isoformX1	*Rhinatrema bivittatum*	Chordata	Amphibia	2 SH2, 1 Kinase
26	A0A6J0SJA2	LOC110072432	*Pogona vitticeps*	Chordata	Reptile	2 SH2, 1 Kinase
27	XP_005281292.1	ZAP-70	*Chrysemys picta bellii*	Chordata	Reptile	2 SH2, 1 Kinase
28	A0A402FYT7	ParPi_0025228	*Paroedura picta*	Chordata	Reptile	2 SH2, 1 Kinase
29	XP_024061102.1	ZAP-70 isoformX1	*Terrapene carolina triunguis*	Chordata	Reptile	2 SH2, 1 Kinase
30	A0A670K6H0	LOC114588403	*Podarcis muralis*	Chordata	Reptile	2 SH2, 1 Kinase
31	A0A6I9XF86	ZAP-70	*Thamnophis sirtalis*	Chordata	Reptile	2 SH2, 1 Kinase
32	H0YPY8	ZAP-70	*Taeniopygia guttata*	Chordata	Bird	2 SH2, 1 Kinase
33	A0A1V4KPZ2	ZAP-70	*Patagioenas fasciata monilis*	Chordata	Bird	2 SH2, 1 Kinase
34	U3I3S8	ZAP-70	*Anas platyrhynchos*	Chordata	Bird	2 SH2, 1 Kinase
35	E1BU42	ZAP-70	*Gallus gallus*	Chordata	Bird	2 SH2, 1 Kinase
36	A0A218UCB5	ZAP-70	*Lonchura striata domestica*	Chordata	Bird	2 SH2, 1 Kinase
37	XP_026721024.1	ZAP-70	*Athene cunicularia*	Chordata	Bird	2 SH2, 1 Kinase
38	XP_029889208.1	ZAP-70	*Aquila chrysaetos chrysaetos*	Chordata	Bird	2 SH2, 1 Kinase
39	XP_040393122.1	ZAP-70	*Cygnus olor*	Chordata	Bird	2 SH2, 1 Kinase
40	KAF1503043.1	ZAP-70	*Megadyptes antipodes*	Chordata	Bird	2 SH2, 1 Kinase
41	U3KHA8	ZAP-70	*Ficedula albicollis*	Chordata	Bird	2 SH2, 1 Kinase
42	A0A3Q0CUC0	LOC101833852	*Mesocricetus auratus*	Chordata	Mammalia	2 SH2, 1 Kinase
43	A0A2K6GLH5	ZAP-70	*Propithecus coquereli*	Chordata	Mammalia	2 SH2, 1 Kinase
44	P43404	ZAP-70	*Mus musculus*	Chordata	Mammalia	2 SH2, 1 Kinase
45	A0A1S3AJ87	LOC103124953	*Erinaceus europaeus*	Chordata	Mammalia	2 SH2, 1 Kinase
46	M3WD79	ZAP-70	*Felis catus*	Chordata	Mammalia	2 SH2, 1 Kinase
47	W5PW03	ZAP-70	*Ovis aries*	Chordata	Mammalia	2 SH2, 1 Kinase
48	G1SPQ8	ZAP-70	*Oryctolagus cuniculus*	Chordata	Mammalia	2 SH2, 1 Kinase
49	H0XG88	ZAP-70	*Otolemur garnettii*	Chordata	Mammalia	2 SH2, 1 Kinase
50	A0A0R4J8U1	ZAP-70	*Rattus norvegicus*	Chordata	Mammalia	2 SH2, 1 Kinase

51	A0A5K1V762	ZAP-70	*Sus scrofa*	Chordata	Mammalia	2 SH2, 1 Kinase
52	A0A2K6R1D7	ZAP-70	*Rhinopithecus roxellana*	Chordata	Mammalia	2 SH2, 1 Kinase
53	F6SWY7	ZAP-70	*Callithrix jacchus*	Chordata	Mammalia	2 SH2, 1 Kinase
54	F7FAI7	ZAP-70	*Macaca mulatta*	Chordata	Mammalia	2 SH2, 1 Kinase
55	A0A2K5MYN1	ZAP-70	*Cercocebus atys*	Chordata	Mammalia	2 SH2, 1 Kinase
56	G1LF11	ZAP-70	*Ailuropoda melanoleuca*	Chordata	Mammalia	2 SH2, 1 Kinase
57	A0A2I3LEH3	ZAP-70	*Papio anubis*	Chordata	Mammalia	2 SH2, 1 Kinase
58	F7AR49	ZAP-70	*Equus caballus*	Chordata	Mammalia	2 SH2, 1 Kinase
59	A0A0D9RXH6	ZAP-70	*Chlorocebus sabaeus*	Chordata	Mammalia	2 SH2, 1 Kinase
60	H2QIE3	ZAP-70	*Pan troglodytes*	Chordata	Mammalia	2 SH2, 1 Kinase
61	G3H1Q5	I79_004087	*Cricetulus griseus*	Chordata	Mammalia	2 SH2, 1 Kinase
62	A0A2K5R1S1	ZAP-70	*Cebus imitator*	Chordata	Mammalia	2 SH2, 1 Kinase
63	A0A6J0E3Y4	LOC102923421	*Peromyscus maniculatus bairdii*	Chordata	Mammalia	2 SH2, 1 Kinase
64	G3QGN8	ZAP-70	*Gorilla gorilla*	Chordata	Mammalia	2 SH2, 1 Kinase
65	G3UMI3	ZAP-70	*Loxodonta africana*	Chordata	Mammalia	2 SH2, 1 Kinase
66	P43403	ZAP-70	*Homo sapiens*	Chordata	Mammalia	2 SH2, 1 Kinase

Table S3: Dissociation constant (K_d) determined from ITC experiment

Construct	K_{d2}	K^*_{d1}
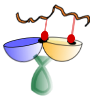 tSH2:ITAM-Y2P-ζ1	3.0 ± 1.4 μM	53.67 ± 4.73 nM
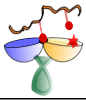 ^{tSH2}R192A:ITAM-Y2P-ζ1	6.1 ± 0.8 μM	---
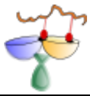 tSH2: ITAM-Y2P-ζ3	8.8 ± 0.8 μM	138.67 ± 3.51 nM
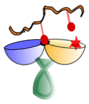 ^{tSH2}R192A:ITAM-Y2P-ζ3	12 ± 0.9 μM	---
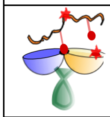 ^{tSH2}R192A: $^{ITAM\text{-}Y2P\text{-}\zeta1}$E13A	9.8 ± 0.75 μM	---

Table S4: Rate parameters used in the model (Corresponding to Figure 2 c-h)

**Rate parameters**	**Values**
k_f	0.35 μM^{-1} s^{-1} (varied for Figure 2c,2d)
$k_b = k_{b1} = k_{b2} = k_{b3}$	0.0007 s^{-1}
k_{close}	0.0875 s^{-1}
k_{open}	0.0007 s^{-1}
k'_{close}	8.75×10^{-8} s^{-1}
k'_{open}	0.0014 s^{-1}
k_1^+	0.00035 μM^{-1} s^{-1}
k_1^-	0.00007 s^{-1}
k_2^+	0.00035 μM^{-1} s^{-1}
k_2^-	0.00007 s^{-1}
k_3^+	0.00035 μM^{-1} s^{-1}
k_3^-	0.7×10^{-9} s^{-1}
w_1	0.0002 (varied for Figure 2f, 2g)
w_2	0.02 (Varied for Figure S2c, S2d)
w_3	Assumed to be same as w_1

Table S5: Rate parameters and corresponding dissociation constants used in the model to match the experimental data of ITAM-Y2P-ζ1 and ITAM-Y2P-ζ3 bindings to t-SH2 domains of ZAP-70 (corresponding to Figure 2i)

**Rate parameters**		**ITAM-Y2P-ζ1**	**ITAM-Y2P-ζ3**
k_f		1.75 μM^{-1} s^{-1}	0.35 μM^{-1} s^{-1}
k_b		0.0035 s^{-1}	0.0007 s^{-1}
k_{close}		0.0875 s^{-1}	0.00875 s^{-1}
k_{open}		0.0035 s^{-1}	0.007 s^{-1}
k'_{close}		8.75×10^{-8} s^{-1}	8.75×10^{-9} s^{-1}
k'_{open}		0.00175 s^{-1}	0.0014 s^{-1}
k_1^+		0.175 μM^{-1} s^{-1}	0.035 μM^{-1} s^{-1}
k_1^-		10.5 s^{-1}	2.1 s^{-1}
k_2^+		0.175 μM^{-1} s^{-1}	0.035 μM^{-1} s^{-1}
k_2^-		10.5 s^{-1}	2.1 s^{-1}
k_3^+		0.00875 μM^{-1} s^{-1}	0.001225 μM^{-1} s^{-1}
k_3^-		3.5×10^{-9} s^{-1}	0.7×10^{-9} s^{-1}
w_1, w_3		10^{-6}	2×10^{-7}
w_2		4×10^{-5}	2×10^{-5}
k_{b1}=k_{b2}=k_{b3}		3.5×10^{-6} s^{-1}	7×10^{-7} s^{-1}
Dissociation Constants	$K_{d1}(=\frac{k_b}{k_f})$	2 nM	2 nM
	K^*_{d1} $(=\frac{k_{b2}}{w_2 k_f})$	50 nM	100 nM
	K_{d2} $(=\frac{k_{b1}}{w_1 k_f})$	2 μM	10 μM
	K_{con} $(=\frac{k_{open}}{k_{close}})$	40 nM	800 nM
	K'_{con} $(=\frac{k'_{open}}{k'_{close}})$	20000	20000

Table S6: Rate parameters used in the model (shown in Figure 3f) to match the experimental data of tSH2: ITAM-YP-ζ1 and ^{tSH2}R39A: ITAM-Y2P-ζ1 (Figure 3 g-h)

<table>
<tr><td colspan="2">Rate parameters</td><td>tSH2: ITAM-YP-ζ1</td><td>^{tSH2}R39A: ITAM-Y2P-ζ1</td></tr>
<tr><td colspan="2">k_f</td><td>0.875 μM^{-1} s^{-1}</td><td>0.4375 μM^{-1} s^{-1}</td></tr>
<tr><td colspan="2">k_b</td><td>0.0035 s^{-1}</td><td>0.0035 s^{-1}</td></tr>
<tr><td colspan="2">k_{close}</td><td>0.875 s^{-1}</td><td>0.875 s^{-1}</td></tr>
<tr><td colspan="2">k_{open}</td><td>0.0875 s^{-1}</td><td>2.1875 s^{-1}</td></tr>
<tr><td colspan="2">$w_1, w_2, w_3, k_{b1}, k_{b2}, k_{b3}, k_1^+, k_1^-, k_2^+, k_2^-, k_3^+, k_3^-, k'_{close}, k'_{open}$ (negligible)</td><td>10^{-10} unit</td><td>10^{-10} unit</td></tr>
<tr><td rowspan="2">Dissociation Constants</td><td>$K_{d1}(=\frac{k_b}{k_f})$</td><td>4 nM</td><td>8 nM</td></tr>
<tr><td>K_{con} $(=\frac{k_{open}}{k_{close}})$</td><td>100 nM</td><td>2.5 μM</td></tr>
</table>

Table S7: Rate parameters used in the model (shown in Figure 6h) to match the experimental data of Syk tSH2 binding (Figure 6i-j). Note, the parameters corresponding to ITAM-YP-ζ1 and ITAM-YP-ζ3 are the same since Syk cannot possibly distinguish between them according to the experiments (Figure 6a, 6i). We only varied $K_d = \frac{k_b}{k_f}$ by changing k_b, and K_d = 60 nM produced the best fit to the data.

Rate symbols	Values<br><br>(both for ITAM-Y2P-ζ1 and ITAM-Y2P-ζ3)
k_f	1.3 μM^{-1} s^{-1}
k_{close}	1.3 s^{-1}
k_b	0.026 s^{-1}, 0.078 s^{-1},0.13 s^{-1}<br><br>($K_d = \frac{k_b}{k_f}$ = 20 nM,60 nM,100 nM)
k_{open} (=k_b)	Assumed to be the same as k_b
$w_{1,} w_2, w_3$	1 (no kinetic penalty)
$k_{b1} = k_{b2} = k_{b3}$(=k_b)	Kept same as k_b
$k_1^+, k_1^-, k_2^+, k_2^-, k_3^+, k_3^-$,<br><br>$k'_{close}$, k'_{open} (negligible)	10^{-10} unit

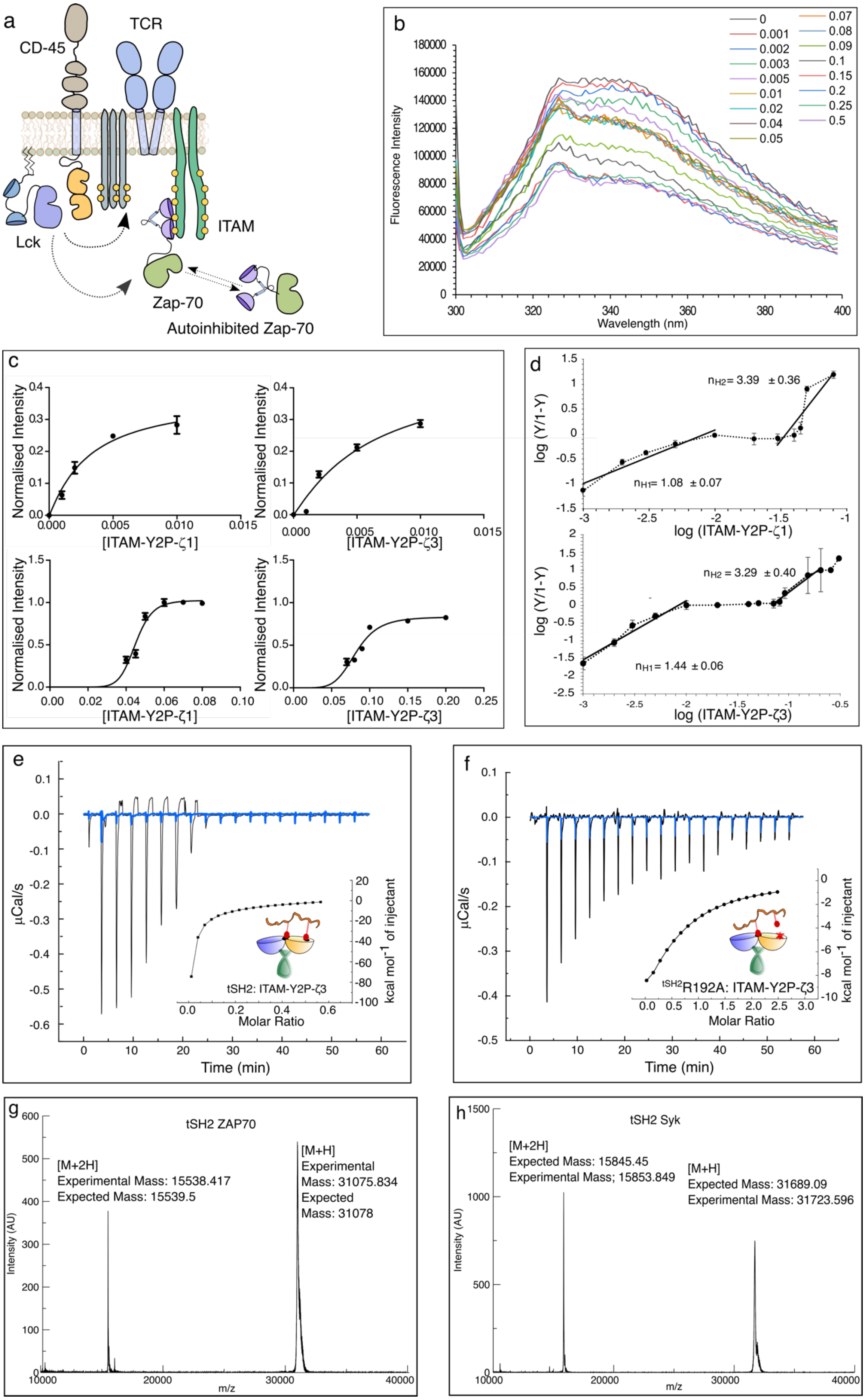
a
CD-45
TCR
Lck
ITAM
Zap-70
Autoinhibited Zap-70
b
Fluorescence Intensity
Wavelength (nm)
c
Normalised Intensity
[ITAM-Y2P-ζ1]
[ITAM-Y2P-ζ3]
d
log (Y/1-Y)
n_{H2}= 3.39 ± 0.36
n_{H1}= 1.08 ± 0.07
log (ITAM-Y2P-ζ1)
n_{H2} = 3.29 ± 0.40
n_{H1}= 1.44 ± 0.06
log (ITAM-Y2P-ζ3)
e
µCal/s
kcal mol^{-1} of injectant
tSH2: ITAM-Y2P-ζ3
Molar Ratio
Time (min)
f
µCal/s
kcal mol^{-1} of injectant
^{tSH2}R192A: ITAM-Y2P-ζ3
Molar Ratio
Time (min)
g
tSH2 ZAP70
[M+2H]
Experimental Mass: 15538.417
Expected Mass: 15539.5
[M+H]
Experimental
Mass: 31075.834
Expected
Mass: 31078
Intensity (AU)
m/z
h
tSH2 Syk
[M+2H]
Expected Mass: 15845.45
Experimental Mass; 15853.849
[M+H]
Expected Mass: 31689.09
Experimental Mass: 31723.596
Intensity (AU)
m/z

Figure S1 Binding of tSH2 domain of ZAP-70 to different ITAM motifs (related to Figure 1): (a) Schematic diagram showing the initiation of T-cell signaling. The Lck Kinase phosphorylates the repeated ITAM motif in the cytosolic domain of T-cell receptor. tSH2 domain of ZAP-70 binds to the doubly phosphorylated ITAM motifs, thus initiates the downstream signaling. (b) Representative fluorescence intensity scanned (λem 300nm to 400nm) from the titration of tSH2 domain of ZAP-70 and ITAM-Y2P-ζ3 peptide. (c) The change in intrinsic fluorescence for tSH2 domain of ZAP-70 with increasing concentration of ITAM-Y2P-ζ1 and ITAM-Y2P-ζ3, respectively (Figure 1d) was fitted to first-order binding equation implemented in the program Prism. (d) Hill plot for the titration of tSH2 domain of ZAP-70 and different peptides (ITAM-Y2P-ζ1 and ITAM-Y2P-ζ3), described in figure 1d. The Hill-coefficient (nH) was determined from the slope of the plot. (e-f) Representative isothermal titration calorimetry for the tSH2 domain and ITAM-Y2P-ζ3 peptide, and ^{tSH2}R192A and ITAM-Y2P-ζ3 peptide, respectively. (g) and (h) MALDI-TOF mass spectroscopy of tSH2 domains of ZAP-70 and Syk, respectively.

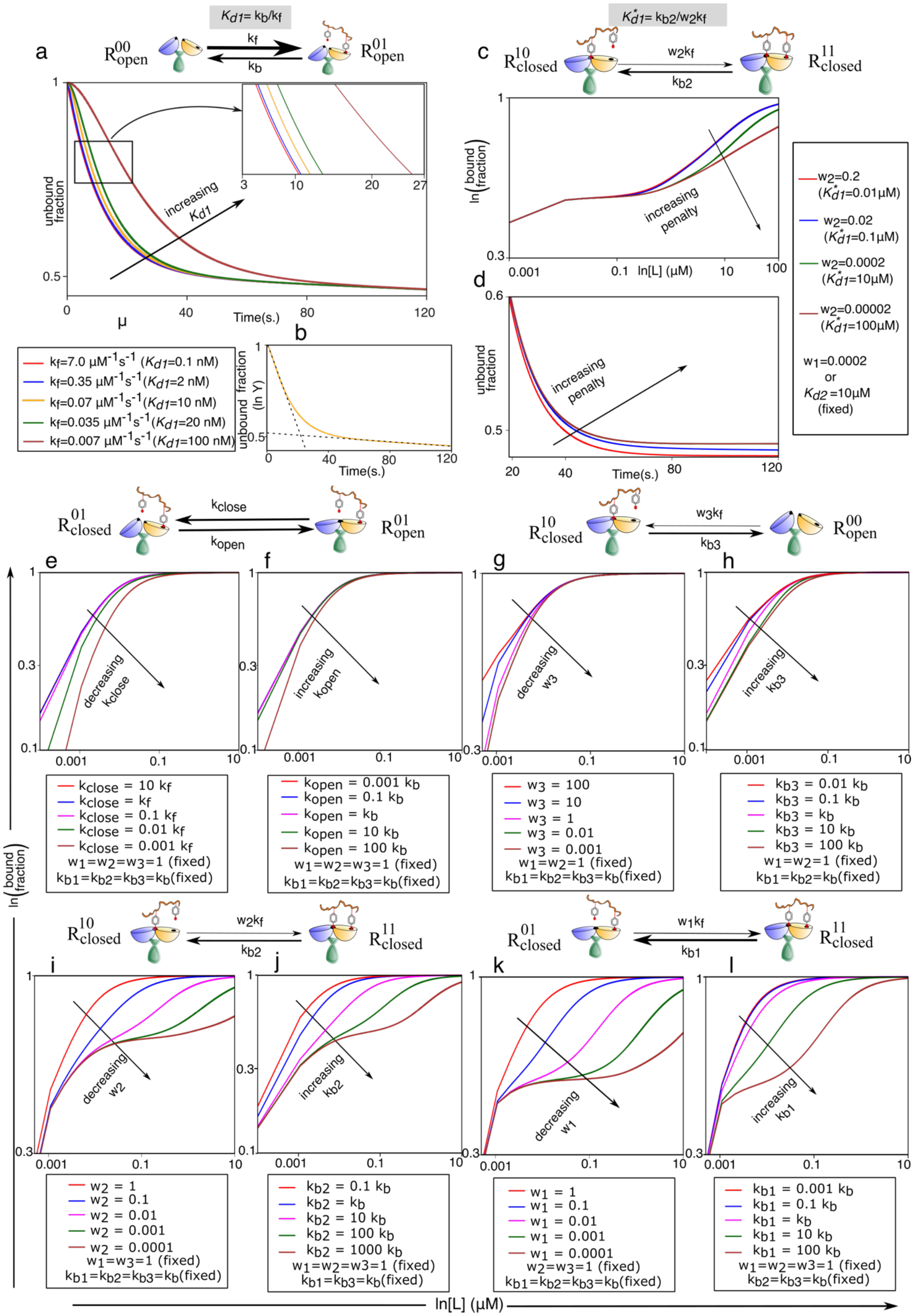

a
K_{d1}= kb/kf
R^{00}_{open}
kf
kb
R^{01}_{open}
unbound fraction
increasing K_{d1}
Time(s.)
b
unbound fraction (ln Y)
Time(s.)
kf=7.0 μM-1s-1 (Kd1=0.1 nM)
kf=0.35 μM-1s-1(Kd1=2 nM)
kf=0.07 μM-1s-1(Kd1=10 nM)
kf=0.035 μM-1s-1(Kd1=20 nM)
kf=0.007 μM-1s-1(Kd1=100 nM)
c
K^*_{d1}= kb2/w2kf
R^{10}_{closed}
w2kf
kb2
R^{11}_{closed}
ln(bound fraction)
increasing penalty
ln[L] (μM)
d
unbound fraction
increasing penalty
Time(s.)
w2=0.2 (K^*_{d1}=0.01μM)
w2=0.02 (K^*_{d1}=0.1μM)
w2=0.0002 (K^*_{d1}=10μM)
w2=0.00002 (K^*_{d1}=100μM)
w1=0.0002 or Kd2 =10μM (fixed)
R^{01}_{closed}
kclose
kopen
R^{01}_{open}
R^{10}_{closed}
w3kf
kb3
R^{00}_{open}
e
decreasing kclose
kclose = 10 kf
kclose = kf
kclose = 0.1 kf
kclose = 0.01 kf
kclose = 0.001 kf
w1=w2=w3=1 (fixed)
kb1=kb2=kb3=kb(fixed)
f
increasing kopen
kopen = 0.001 kb
kopen = 0.1 kb
kopen = kb
kopen = 10 kb
kopen = 100 kb
w1=w2=w3=1 (fixed)
kb1=kb2=kb3=kb(fixed)
g
decreasing w3
w3 = 100
w3 = 10
w3 = 1
w3 = 0.01
w3 = 0.001
w1=w2=1 (fixed)
kb1=kb2=kb3=kb(fixed)
h
increasing kb3
kb3 = 0.01 kb
kb3 = 0.1 kb
kb3 = kb
kb3 = 10 kb
kb3 = 100 kb
w1=w2=1 (fixed)
kb1=kb2=kb(fixed)
ln(bound fraction)
R^{10}_{closed}
w2kf
kb2
R^{11}_{closed}
R^{01}_{closed}
w1kf
kb1
R^{11}_{closed}
i
decreasing w2
w2 = 1
w2 = 0.1
w2 = 0.01
w2 = 0.001
w2 = 0.0001
w1=w3=1 (fixed)
kb1=kb2=kb3=kb(fixed)
j
increasing kb2
kb2 = 0.1 kb
kb2 = kb
kb2 = 10 kb
kb2 = 100 kb
kb2 = 1000 kb
w1=w2=w3=1 (fixed)
kb1=kb3=kb(fixed)
k
decreasing w1
w1 = 1
w1 = 0.1
w1 = 0.01
w1 = 0.001
w1 = 0.0001
w2=w3=1 (fixed)
kb1=kb2=kb3=kb(fixed)
l
increasing kb1
kb1 = 0.001 kb
kb1 = 0.1 kb
kb1 = kb
kb1 = 10 kb
kb1 = 100 kb
w1=w2=w3=1 (fixed)
kb2=kb3=kb(fixed)
ln[L] (μM)

Figure S2 (Related to Figure 2): Effects of parameter variation on the binding kinetics in our model. (a) Representative kinetic profiles of the unbound fraction for different K_{d1} values (indicated in the box below). The curves are produced in the pre-steady state with initial concentrations of R^{00}_{open}=500 nM and L=10 μM. The inset shows a close-up view of a portion of the curves highlighting the trend of K_{d1} variation. (b) Kinetics exhibit two-step decay with two distinct exponential fits (dashed line) in semi-log plot. (c) The Effect of K^{*}_{d1} variation (by changing the penalty factor w_2) on the bound fraction in the steady state. (d) Effects of K^{*}_{d1} variation on kinetics of the unbound fraction showing only a single-step decay. Together, (c-d) shows that the variation of K^{*}_{d1} does not significantly affect the plateau width and the kinetic profiles in the steady state and pre-steady state conditions, respectively (keeping K_{d1}= 2 nM and K_{d2}=K_{d3}=10 μM). (e-h) The steady-state response against the ligand concentration does not show biphasic behaviour when the transition rates of the partially-bound closed states (R^{01}_{closed} or R^{10}_{closed}) to the open state were varied. (i-l) When the transition rates from the partially-bound closed states (R^{01}_{closed} or R^{10}_{closed}) to final *holo*-state (R^{11}_{closed}) were varied, biphasic curves were obtained. For the panels e-l, the respective rate parameters which were varied are shown in the boxes below, while kinetic penalties were removed from other steps. Other parameters are as in Table S4.

a

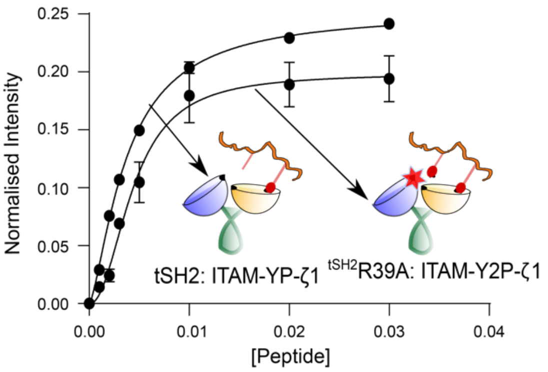

b

CONSTRUCT	K_d	nH
tSH2R39A: ITAM-Y2P-ζ1	8 ± 1.05 nM	1.3 ± 0.12
tSH2: ITAM-YP-ζ1	3.7 ± 0.1 nM	1.44 ± 0.01

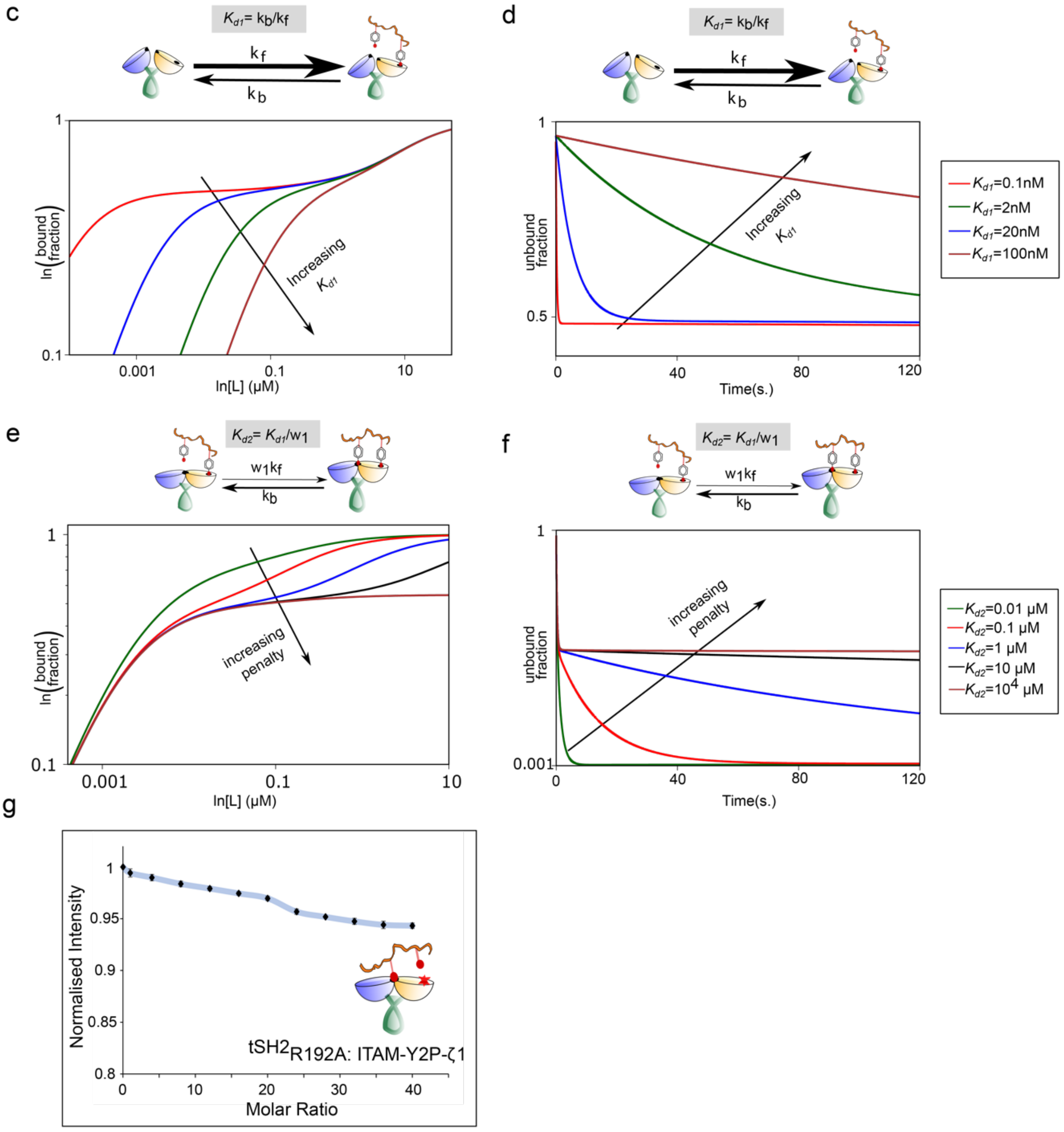

Figure S3 (Related to Figure 3): (a) The change in intrinsic fluorescence for ^{tSH2}R39A: ITAM-Y2P-ζ1 and tSH2 wildtype: ITAM-YP-ζ1 titration, respectively, was fitted to first-order binding equation implemented in the program Prism. (b) Tabulation of the K_d and Hill coefficient (nH) of the binding of ^{tSH2}R39A and ITAM-Y2P-ζ1, and tSH2 wildtype and ITAM-YP-ζ1, respectively. (c-d) Effects of K_{d1} variation in the reduced model shown in Figure 3k. Similar to the results of the full model (Figure 2c-h), the K_{d1} determines the sensitivity of the bound-fraction in the steady-state (panel c) and modulates the initial decay in the kinetics of unbound fraction (panel d). (e) Increasing K_{d2} broadens the plateau width in the steady state, similar to the full model. (f) Binding kinetics again show a two-step decay as in the full model. (g) The change in intrinsic fluorescence is plotted against the indicated ligand to protein ratio during the titration of 0.5 μM ^{tSH2}R192A with the ITAM-Y2P-ζ1. The titration was performed up to 20μM of ITAM-Y2P-ζ1.

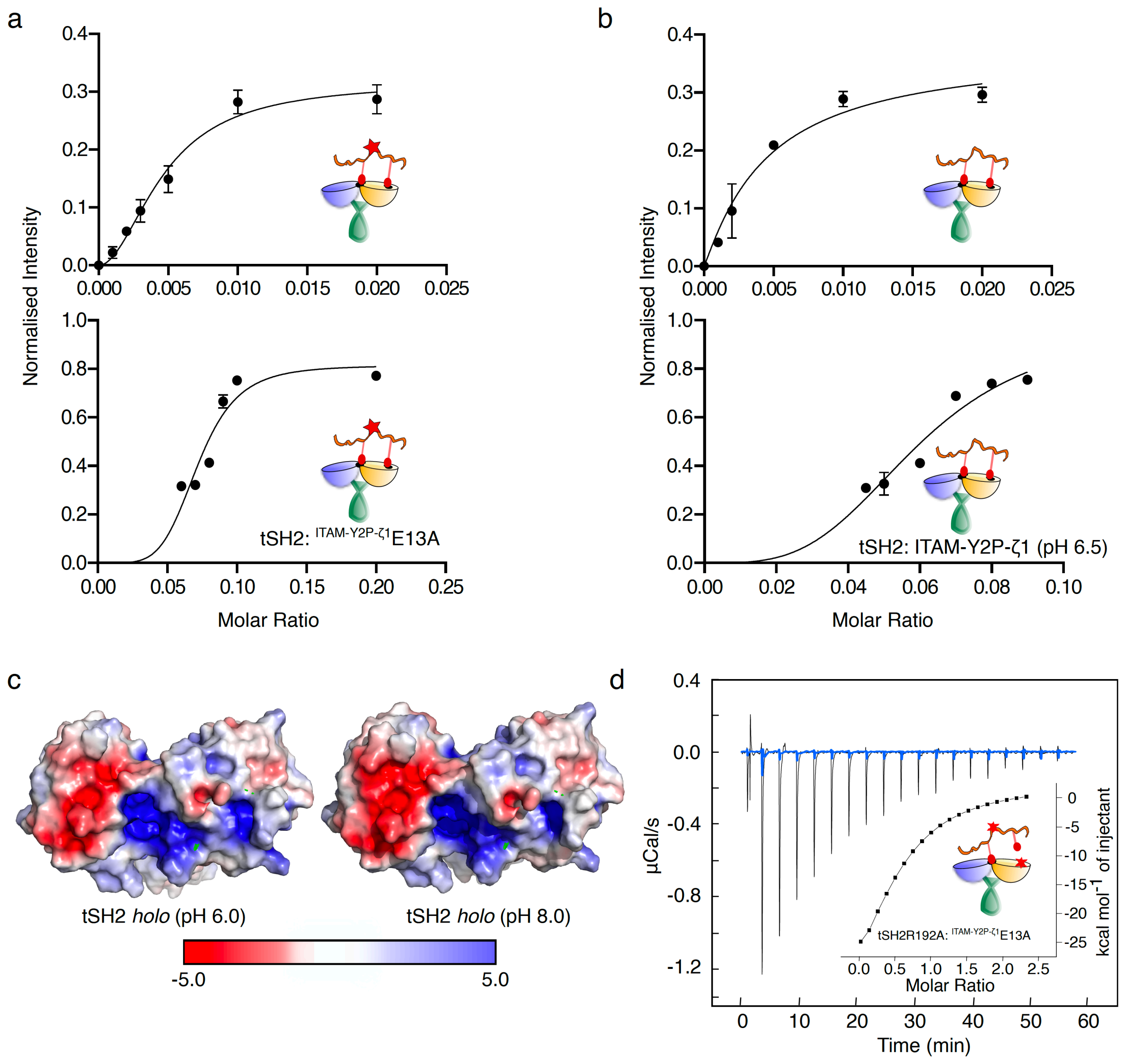

Figure S4 (Related to Figure 4): (a-b) The change in intrinsic fluorescence for tSH2 domain of ZAP-70: $^{\text{ITAM-Y2P-}\zeta1}$E13A at pH 8.0, and ITAM-Y2P-ζ1 at pH 6.5, respectively, was fitted to first-order binding equation implemented in the program Prism. (c) Surface potential for the tSH2 *holo* structure of ZAP-70 (PDB ID: 2OQ1) at pH 6.0 and pH 8.0, respectively, are shown. Color bar represents charge density. (d) Representative isothermal titration calorimetry for the $^{\text{tSH2}}$R192A and $^{\text{ITAM-Y2P-}\zeta1}$E13A peptide binding.

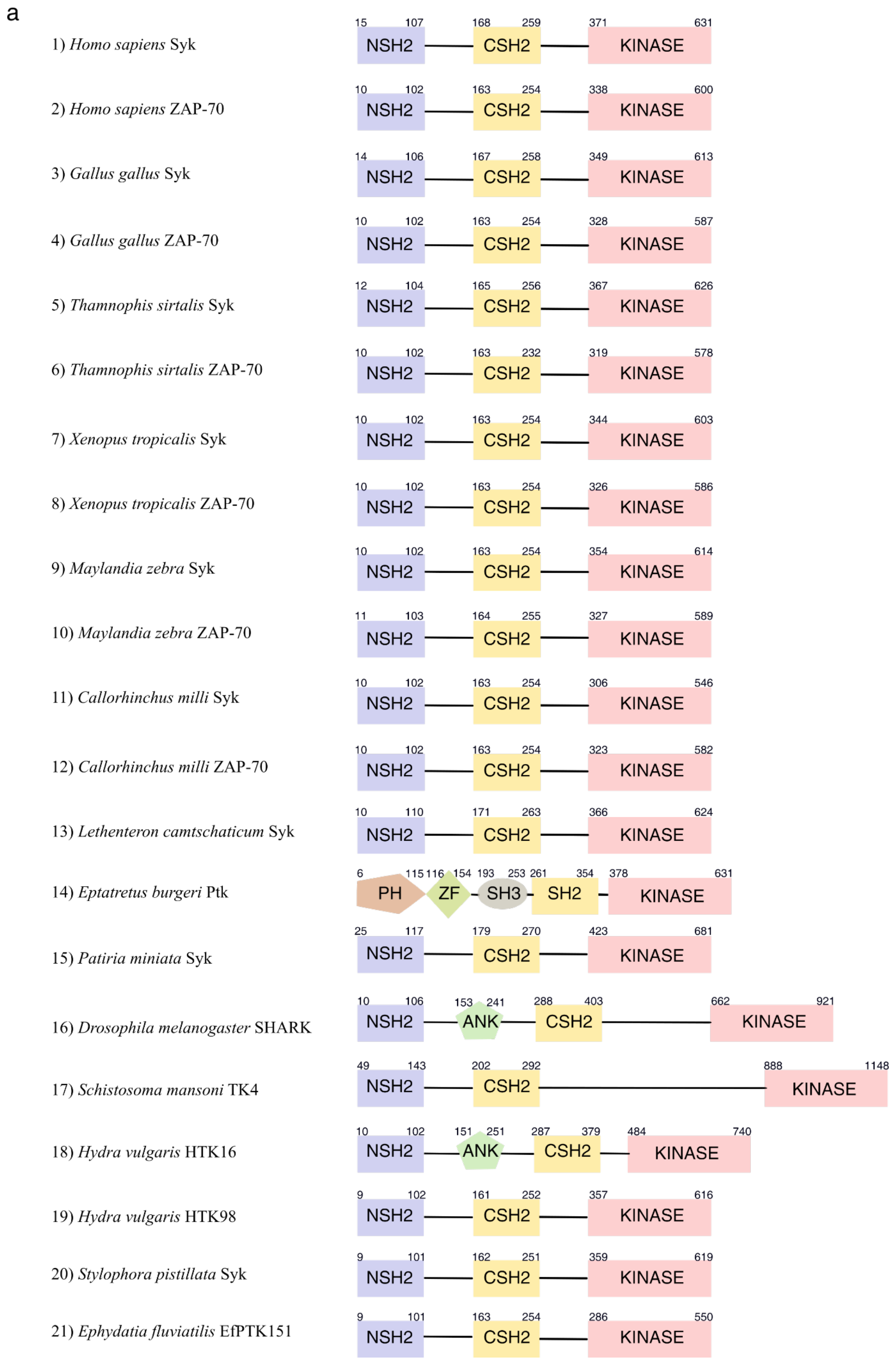

Figure S5: Domain Architecture of Syk Family Kinases (Related to Figure 5)

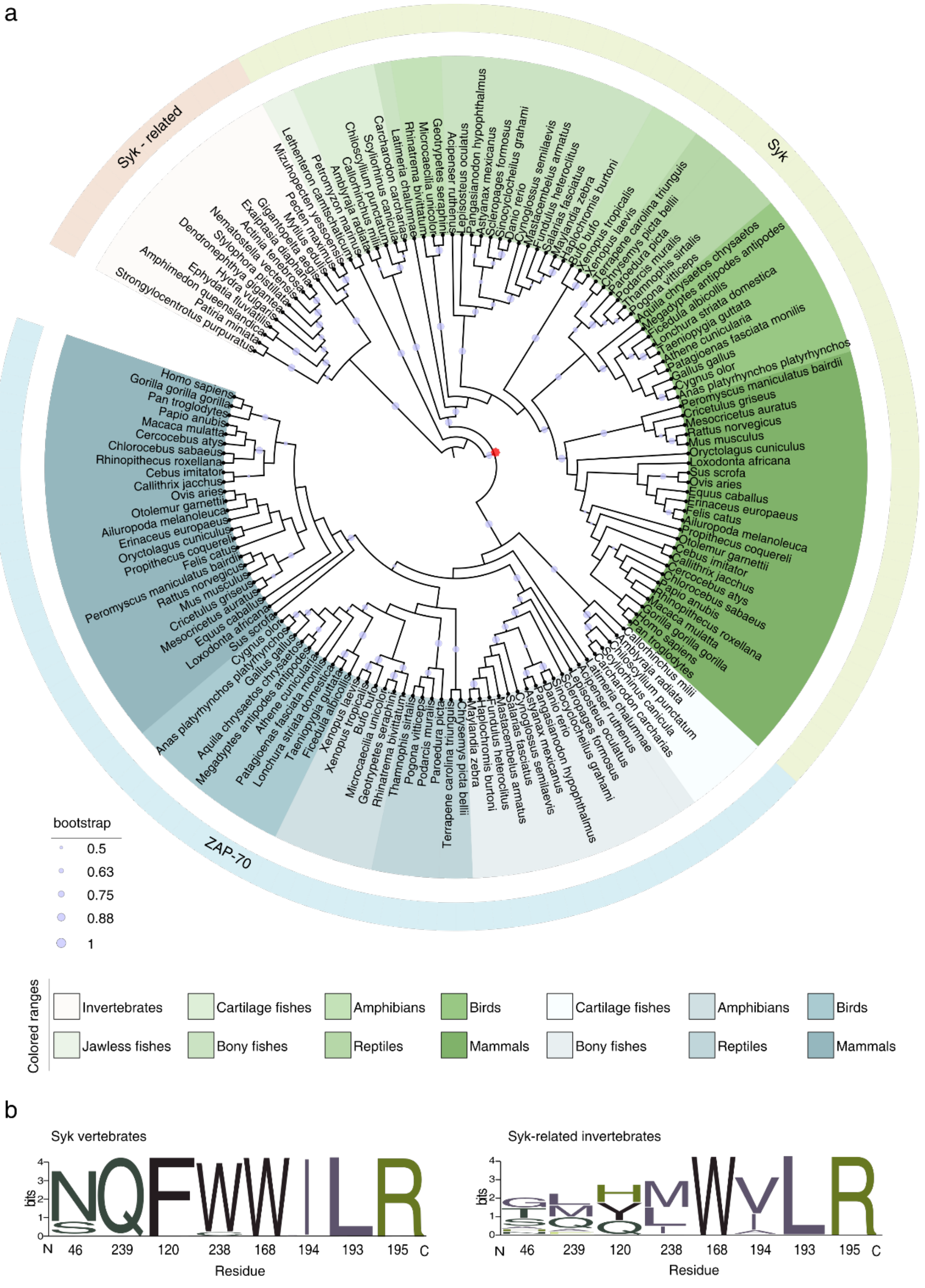

Figure S6: (Related to Figure 5): (a) The phylogenetic tree represents the evolutionary relationship between Syk, Syk-related kinases, and ZAP-70. The color code represents the classes to which each species belongs. The red dot depicts the emergence of ZAP-70 in jawed vertebrates from cartilage fish to mammals. (b) Sequence logo showing the conservation of

network residues of Syk in vertebrates(left) and invertebrates(right). Except for N46, and W238 all other residues are conserved in vertebrates. The Syk-related kinases show high residue variation in invertebrates.

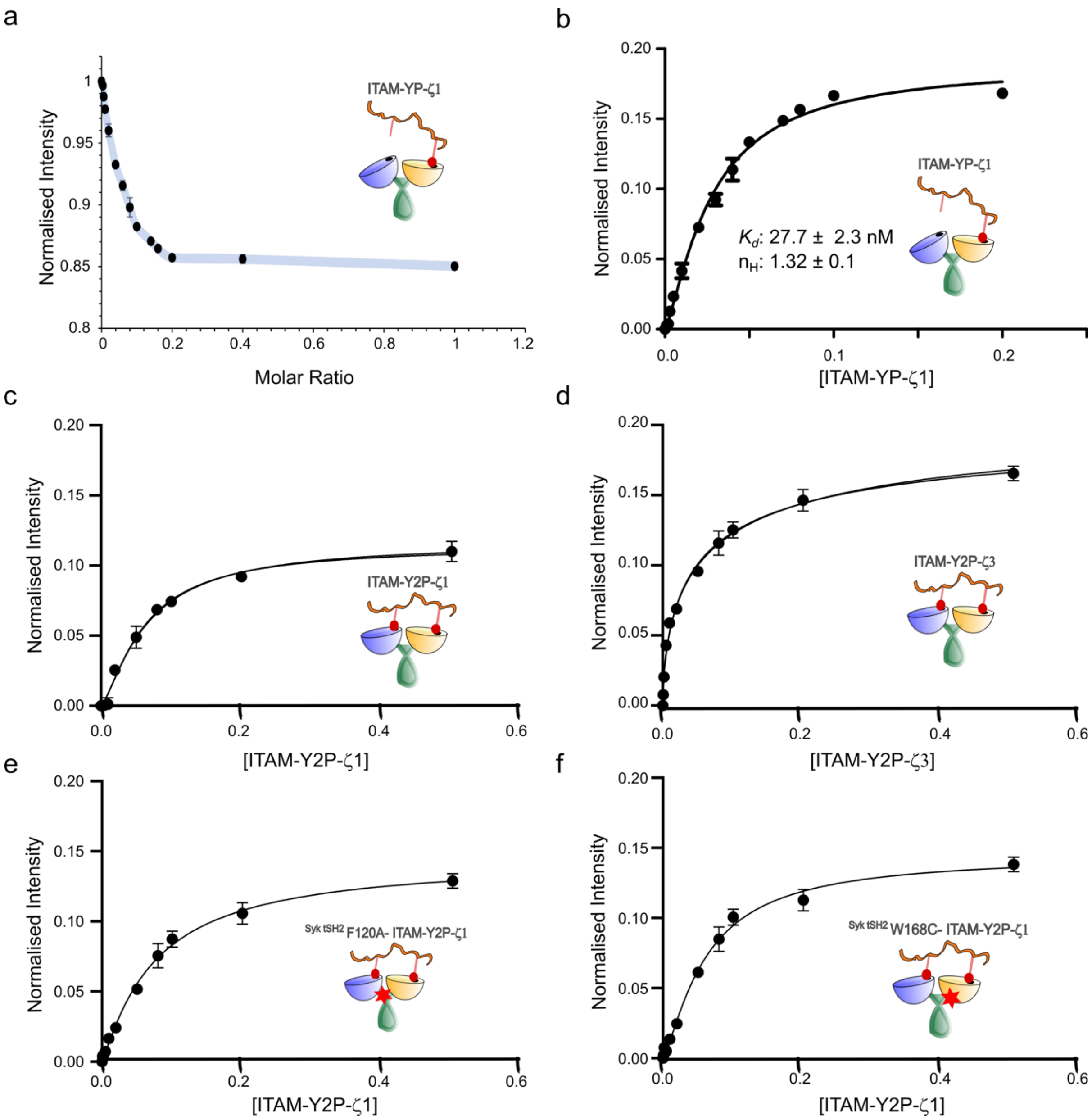

Figure S7: Binding of Syk tSH2 domain and mutants and doubly-phosphorylated ITAM-Y2P peptides (Related to Figure 6): (a-b) Titration of ITAM-YP-ζ1 and tSH2 domain of Syk determined from the measurement of intrinsic tryptophan-fluorescence at the indicated ligand

to protein molar ratio. The error bar represents the standard deviation from three experiments. (c- d) The change in intrinsic fluorescence for tSH2 domain of Syk with increasing concentration of ITAM-Y2P-ζ1and ITAM-Y2P-ζ3 (Figure 5a) was fitted to first-order binding equation implemented in the program Prism. (e-f) Syk tSH2 mutants (F120A and W168C) was titrated with increasing concentration of ITAM-Y2P-ζ1 and the data was fitted to first-order binding equation using Prism.
